# Single–Atom Iron Sites Enable Enhanced Arsenic Removal From Water Through Synergistic Adsorption–Oxidation

**DOI:** 10.1002/advs.76858

**Published:** 2026-07-29

**Authors:** Tao Sun, Chao Wang, Shihang Wu, Xiaojia Zhou, Penggang Pei, Huijuan Yu, Yun Zhang, Qingqing Huang, Yuebing Sun

**Affiliations:** ^1^ Key Laboratory of Original Environmental Pollution Prevention and Control Ministry of Agriculture and Rural Affairs/Tianjin Key Laboratory of Agro‐Environment and Agro‐Products Agro‐Environmental Protection Institute Ministry of Agriculture and Rural Affairs Tianjin China; ^2^ Department of Life Sciences Changzhi University Changzhi China

**Keywords:** adsorption–oxidation, arsenic, single–atom iron, structure modulation, synergistic mechanism

## Abstract

Iron (Fe)–based adsorbents represent a transformative approach for arsenic (As) decontamination, yet achieving atomic–level control of Fe species for efficient As(III) adsorption–oxidation remains a formidable challenge. This study introduces single–atom Fe–decorated nitrogen–rich porous biochar (SAFeC), a breakthrough material with dual As(III) adsorption–oxidation capabilities. SAFeC achieves unprecedented performance, with an adsorption capacity of 237.5 mg g^−1^ and 56.9% of the immobilized As species existing as As(V), while exhibiting excellent environmental robustness and recyclability. Fe atoms in SAFeC display outstanding chemical reactivity toward As(III) (39833.54 mg (g·Fe)^−1^). Atomic–scale characterization and theoretical calculations reveal that SAFeC captures As(III) from water through monodentate inner‐sphere complexation, precipitation, hydrogen bonding, and electrostatic attraction. The complementary synergy between support and single–atom Fe facilitates the activation of molecular oxygen, generating ^1^O_2_ as the primary oxidant for As(III) transformation. This study not only underscores the exceptional efficacy of single–atom Fe active sites in the advanced purification of As(III) but also paves the way for the development of next–generation environmental remediation materials to tackle global As contamination challenges.

## Graphical abstract

Single–atom iron anchored on nitrogen–doped porous biochar exhibited ultra–efficient performance, exceptional environmental resilience, and integrated adsorption–oxidation functionality. The multifunctional architecture of the support and the unparalleled reactivity of single–atom iron underpinned the cooperative adsorption and oxidation of As(III). This work advances the environmental application of single–atom iron sites and offers a sustainable blueprint for tackling the global As contamination challenge.

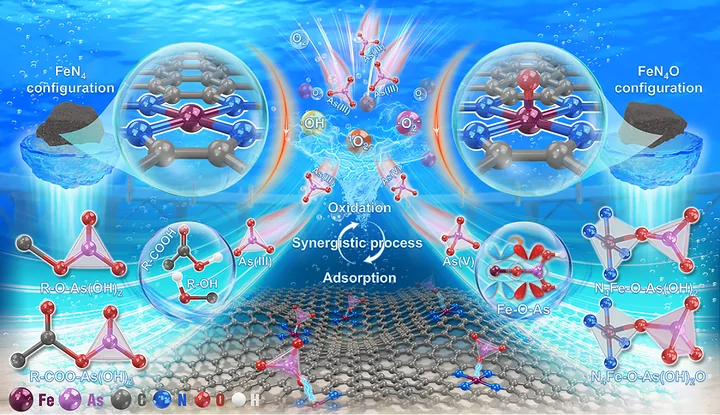

## 1 Introduction

Arsenic (As) contamination is widely acknowledged as a critical global environmental and public health challenge. In natural water systems, As primarily exists as arsenite (As(III)) and arsenate (As(V)), with As(III) being more toxic and mobile due to its higher solubility and bioavailability [1]. Geological release is the primary source of As contamination in water, while human activities, including mining, industrial processes, and the overuse of pesticides and fertilizers, have considerably elevated As levels in aquatic environments [2]. Alarmingly, an estimated 200 million people worldwide are exposed to high–As groundwater, placing them at risk of severe health complication [3]. Consequently, the World Health Organization (WHO) has classified As as one of the most hazardous environmental toxicants. Prolonged As exposure not only threatens ecosystems and human well–being, but also hinders progress toward achieving the UN Sustainable Development Goals.

To mitigate As pollution, various decontamination strategies, including adsorption, ion exchange, membrane filtration, electrochemical treatment, and precipitation, have been widely employed [4, 5]. Among these, adsorption stands out as a highly promising approach due to its cost–effectiveness, superior performance, and operational simplicity. In particular, various iron (Fe)–based adsorbents, such as iron (hydro)oxides (FeO) and zero–valent iron (ZVI), have gained significant attention owing to the strong chemical bonding interactions between Fe and As [6, 7, 8]. However, the interaction mechanisms between various Fe species and As differ considerably. The limited electrical conductivity and sluggish redox kinetics of FeO restrict their effectiveness in As(III) oxidation [9], whereas FeO typically exhibits greater affinity for As(V) through inner–sphere complexation [10]. Conversely, ZVI displays stronger surface affinity toward As(III) than As(V) [11]. During the oxidation of ZVI, reactive intermediates are generated that promote As(III) oxidation [12], while the in–situ formed FeO subsequently immobilizes As(V) via electrostatic attraction, surface complexation, and precipitation [13]. Moreover, continuous ZVI corrosion provides a steady supply of reactive sites for As(III) fixation [14]. Despite these advantages, several limitations remain, including the difficulty in controlling Fe particle size and morphology during synthesis, aggregation and passivation of nano‐ZVI, and potential Fe leaching that compromises both performance and environmental safety [15]. Because As(III) mainly exists as neutral H_3_AsO_3_ under near‐neutral conditions, its direct adsorption is more difficult than that of As(V). Thus, oxidation–adsorption coupled systems have attracted increasing attention, including chemical, photo–driven, electrochemical, and reactive Fe–based processes. For instance, ultraviolet C (UVC)/H_2_O_2_– or UVC/NaOCl–driven oxidation coupled with ZnAl–layered double hydroxide adsorption has been reported for drinking–water As removal [16]. However, such systems generally require external oxidants, UV irradiation, electrical input, and/or separate adsorption units, leading to increased operational complexity and cost. Therefore, considering the high mobility, toxicity, and widespread occurrence of As(III), designing integrated Fe–based materials that can simultaneously promote As(III) oxidation and arsenic immobilization under mild conditions remains highly desirable.

The concept of single–atom catalysts (SACs) has served as a key inspiration for the development of innovative Fe–based remediation materials. Isolated metal single–atom sites maximize atomic utilization and minimize metal consumption [17]. Strong metal–support interactions facilitate charge transfer, which significantly boosts the reactive activity and structural stability [18, 19]. However, because isolated atoms possess high surface free energy, suitable supports with abundant anchoring sites are necessary to prevent migration and aggregation [20]. Biochar, characterized by its porous structure, abundant active sites, and environmental sustainability, is considered an excellent candidate for stabilizing single metal atoms [21]. Wang et al. anchored single Fe atoms on furfural–residue biochar to achieve highly efficient degradation of sulfamethoxazole [22]. Building on this concept, Zhou et al. grafted Fe–N moieties onto shrimp–shell biochar to construct a Fe single–atom catalyst that activated peroxymonosulfate (PMS) via mediated electron transfer, enabling rapid aniline removal (94.5% within 5 min) [23]. Xiong et al. further demonstrated that Fe single atoms dispersed on Enteromorpha–derived biochar acted as catalytic sites for PMS adsorption and activation, subsequently generating singlet oxygen for efficient imidacloprid degradation [24]. Despite these promising advances, the potential of single–atom Fe–decorated biochar for As–contaminated water treatment and the associated mechanistic pathways remain largely unexplored.

By 2025, China's annual crayfish production is projected to exceed 3.4 million tons (China Crayfish Industry Development Report 2025), resulting in substantial shell waste from the food industry. Crayfish shell (CS) is particularly attractive as a biochar precursor because it is abundant, low‐cost, and rich in chitin–a biopolymer containing amino groups that act as intrinsic nitrogen (N) sources for in–situ N doping during carbonization [25]. Our previous research demonstrated that O/N–containing functional groups on crayfish shell–derived biochar effectively captured As(III/V) through electrostatic attraction and surface complexation [26]. Notably, the intrinsic N atoms in CS can coordinate with Fe atoms to form stable Fe–N coordination structures, which anchor and stabilize atomically dispersed Fe species [27]. Moreover, the electronegativity difference between Fe and N induces electron cloud redistribution, optimizing the Fe atom electronic structure and enhancing reactivity [28]. Building on these insights, we implemented an innovative “waste–to–remedy” strategy by converting N–rich kitchen waste (CS) into a structurally multifunctional carbon support. Through a cost–effective and scalable synthesis route, we successfully anchored single–atom Fe sites onto this N–enriched substrate, creating a novel single–atom Fe decorated N–rich porous biochar (SAFeC). The atomic dispersion and coordination environment of Fe atoms were confirmed via AC‐HAADF–STEM and XAS analyses. The synergistic combination of highly active single–atom Fe centers and an outstanding support structure endowed SAFeC with exceptional As removal and oxidation capability, together with remarkable stability and anti–interference properties. The integration of multiple characterization techniques and theoretical calculations further elucidated the quantitative structure–activity relationships and underlying mechanisms. This study provides pioneering insights into the design of remediation materials for As–contaminated water and advances the mechanistic understanding of As interactions with single–atom Fe–based biochars.

## 2 Results and Discussion

### 2.1 Material Synthesis and Characterization

We synthesized the SAFeC using a modified, cost–effective, and environmentally friendly two–step pyrolysis method [29]. Initially, crayfish shell (CS) was pyrolyzed to produce biochar, which served as the N–rich support material. The CS–derived biochar was mixed with an Fe–containing solution, freeze–dried, and subjected to secondary pyrolysis for synthesizing the SAFeC. This synthesis strategy leverages the abundant N–rich composition of CS [30]. Anaerobic pyrolysis of CS yielded N–rich biochar endowed with abundant surface functional groups, rich defects, and exceptional porous structures [31], thereby providing effective binding sites for anchoring Fe atoms [19]. The secondary annealing step further stabilized the Fe atoms by forming new chemical bonds and enhancing the interaction between the Fe atoms and the support [32].

The Fe content of SAFeC ranged from 0.39% to 5.25%, consistent with the targeted Fe loading (Figure S2). Fe incorporation significantly improved the surface morphology and porosity of SAFeC, as evidenced by EDS imaging showing uniform Fe distribution (Figure S3). Quantitative pore analysis exhibited a specific surface area (SSA) of 29.43–76.04 m^2^ g^−1^, 2.31–17.73 times higher than that of NC (4.29–11.67 m^2^ g^−1^) (Figures S4 and S5). Additionally, SAFeC exhibited greater total pore volume (TPV) (0.14–0.24 cm^3^ g^−1^ vs. 0.02–0.08 cm^3^ g^−1^ for NC), and an average pore diameter (APD) of 7.46–13.24 nm, confirming its mesoporous features. The increased surface area and pore volume are mainly attributed to Fe‐assisted etching during the loading process, which introduced heterogeneous pores and partially removed impurities within the carbon support [33, 34]. The improved porous structure provided abundant anchoring sites that facilitated the uniform dispersion and stabilization of single Fe atoms [35], while simultaneously increasing the exposure of active sites, and enhancing interfacial electron and mass transfer [36, 37], thereby promoting efficient As(III) fixation [38].

The structural characteristics of SAFeC were comprehensively investigated using XRD, FTIR, Raman, EPR, and XPS. XRD patterns showed no diffraction peaks for crystalline Fe (metallic Fe or oxides) in any samples (Figure S6), implying isolated atomically dispersed Fe. Raman spectra revealed that the integrated area ratios of the D–band and G–band (I_D_/I_G_) were 3.21–4.07, 3.66–4.14, and 3.58–4.34 for C3FeY, C5FeY, and C7FeY, respectively (Figure S7). The lower I_D_/I_G_ of C5FeY and C7FeY compared to their corresponding supports indicated a lower defect degree, likely due to the embedding of Fe atoms within the defects. This was corroborated by EPR (Figure S8), which showed decreased vacancies and unpaired electrons after Fe loading. FTIR spectra exhibited typical peaks of —OH, C = O, C—O, and aromatic C—C stretching vibrations in CXFeY, along with a weak Fe —O band at 570 cm^−1^ (Figure S9). The 2D‐COS analysis of FTIR spectra provided insights into the evolution of surface functional groups in CXFeY induced by Fe loading. The (a)synchronous map signals revealed that, upon Fe loading, the responsiveness of the functional groups in C3FeY, C5FeY, and C7FeY was ranked as CO_3_^2−^ > Fe —O > C —C > C —O > C = O, CO_3_^2−^ > C —C > Fe —O > C —O > C = O, and Fe —O > C —O > C —C > CO_3_^2−^ > C = O, respectively. Furthermore, the sequence of functional group change for C3FeY, C5FeY, and C7FeY was determined as: C —O > CO_3_^2−^> Fe —O > C = O > C —C, C —C > C = O > CO_3_^2−^ > Fe —O > C —O, and C = O > CO_3_^2−^ > C —O > C —C > Fe —O, respectively (Tables S1–S3). XPS further confirmed chemical states in CXFeY. C1s spectra indicated predominant O —C = O (10.83%–26.00%), C —O (8.72%–50.31%), and aromatic C —C (32.15–70.62%) bonds, with an increase in the aromatization degree during the synthesis process (Figure S10). Fe 2p spectra displayed distinct Fe(II)/Fe(III) signals with intensity increasing proportionally to Fe loading, while no metallic Fe was detected (Figure S11). O 1s spectra exhibited weak Fe‐O contributions (4.19%–15.86%) (Figure S12). N 1s spectra of NC consisted of graphitic—N, pyrrolic—N, and pyridinic—N (Figure S13). Upon Fe incorporation, the graphitic—N content increased, a Fe—N signal (∼399 eV) emerged, and the pyridinic—N binding energy shifted, indicating Fe coordination with pyridinic—N, accompanied by electron transfer from Fe to the NC framework [36].

The electrochemical properties of SAFeC were evaluated using Zeta potential, electron exchange capacity (EEC), cyclic voltammetry (CV) curves, Tafel curves, and electrochemical impedance spectroscopy (EIS). Fe loading increased the pH_pzc_ of the materials (Figure S14). The Nyquist plots revealed that the charge–transfer resistance (Rct) of C3FeY (54.5–76.5 Ω) and C5FeY (64.9–181.1 Ω) was markedly lower than that of their corresponding supports C3 (116.2 Ω) and C5 (276.2 Ω), respectively, indicating enhanced interfacial electron transport (Figure S15). Correspondingly, Fe incorporation improved the electron–accepting capacity (EAC) and EEC of C3FeY and C5FeY (Figure S16). With increasing Fe loading, C5FeY demonstrated higher specific capacitance (SCP), corrosion current (CC), and corrosion potential (CP) than C5 (Figures S17 and S18), reflecting accelerated electron transfer. Given that high conductivity promotes ROS generation [39], these results imply that C5FeY possesses superior As(III) adsorption–oxidation capacity.

Considering the above characterization and adsorption–oxidation performance (see next section), C5Fe0.5 and C5Fe2 were selected as representative materials and further probed by AC–HAADF–STEM and XAS to elucidate the Fe microenvironment at the atomic scale. TEM and HRTEM images disclosed no Fe clusters or nanoparticles in C5Fe0.5 and C5Fe2 (Figure 1a–c,f–h). AC–HAADF–STEM confirmed atomic–level Fe dispersion, visible as isolated bright spots (Figure 1d,i), echoing the XRD findings. EDS mapping further verified the homogeneous distribution of C, O, N, and Fe across the carbon architecture (Figure 1e,j). XANES spectra displayed features resembling FePc, particularly the pre–edge peak around 7114 eV, characteristic of an Fe—N_4_ square–planar coordination (Figure 1k). The absorption edges of C5Fe0.5 and C5Fe2 were situated between FeO and Fe_2_O_3_, overlapping with FePc, indicating an Fe oxidation state between +2 and +3. Notably, Fe in C5Fe2 displayed a higher average valence than in C5Fe0.5, consistent with XPS observations. FT–EXAFS spectra exhibited a prominent peak at ∼1.48 Å, attributed to the Fe —N/O configuration, while no Fe–Fe peak at 2.18 Å confirmed the absence of metallic aggregation (Figure 1l). EXAFS fitting indicated coordination numbers of 3.6 and 5.1 for Fe in C5Fe0.5 and C5Fe2, with average bond lengths of 1.968 and 1.957 Å, respectively, close to FePc (2.017 Å) (Table S4). WT analysis revealed a maximum intensity at 4 Å^−1^, consistent with Fe—N coordination, with no Fe—Fe peak at 7.46 Å^−1^, further corroborating isolated Fe single‐atom sites (Figure 1n). Based on the above results and previous studies [40, 41], the most plausible configurations for Fe atoms in C5Fe0.5 and C5Fe2 were Fe —N_4_ and Fe —N_4_O, respectively.

**FIGURE 1:**
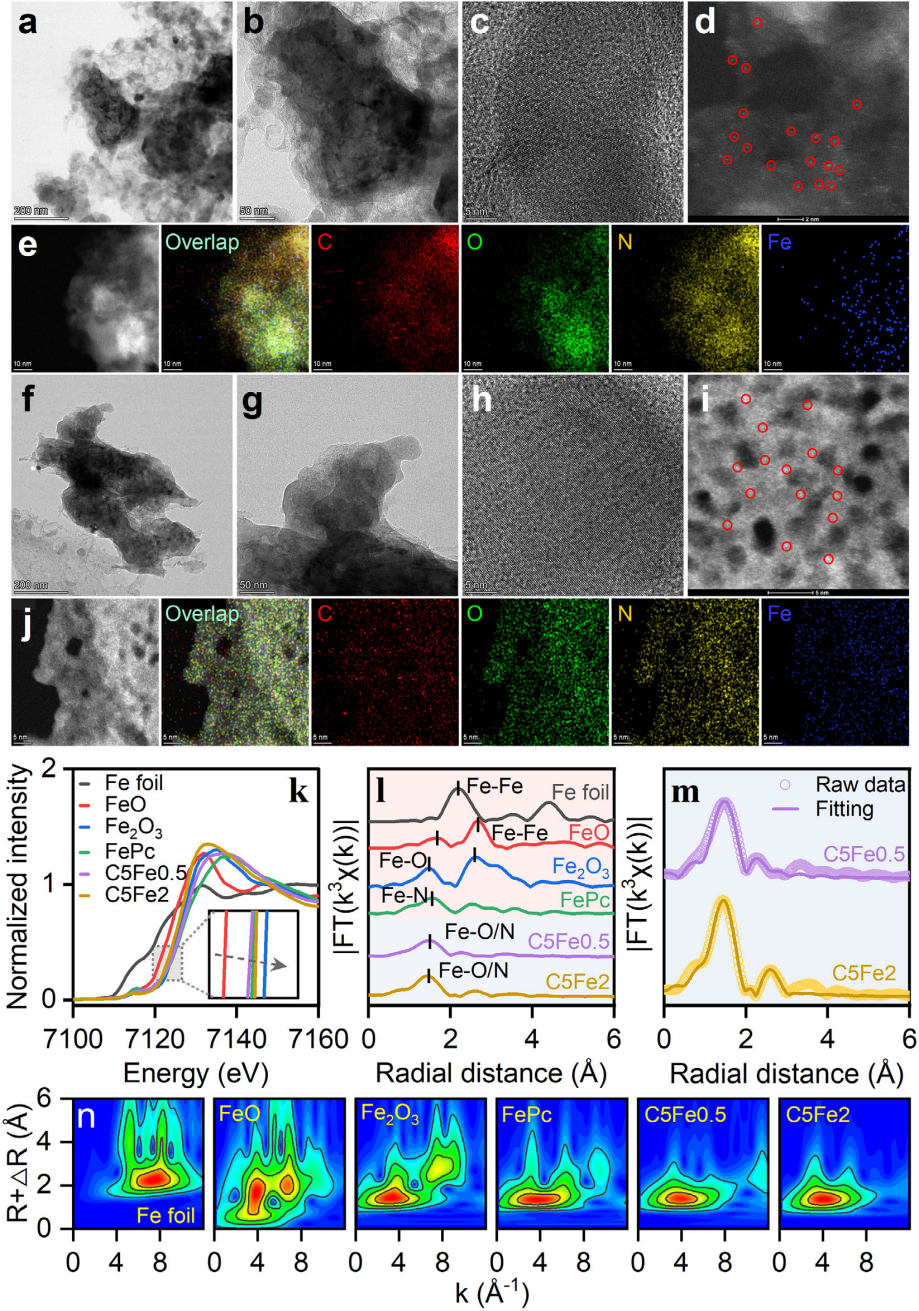
TEM, HRTEM, AC‐HAADF‐STEM, and EDS mappings of (a–e) C5Fe0.5 and (f–j) C5Fe2. (k) Normalized Fe K‐edge XANES spectra, (l) FT‐EXAFS spectra, (m) EXAFS fitting, and (n) WT analysis of references, C5Fe0.5, and C5Fe2.

### 2.2 As(III) Adsorption–‐Oxidation Performance

As illustrated in Figure 2a, pristine NC exhibited negligible As(III) removal, whereas Fe loading significantly improved its affinity toward As(III). Both C3FeY and C5FeY achieved rapid As(III) removal within 30 min, while C7 with 0.5% Fe doping showed unexpectedly higher performance than samples with higher Fe contents. Adsorption kinetics and isotherm experiments revealed that Fe incorporation substantially accelerated the adsorption rate and increased the maximum adsorption capacity (Figure 2b,c). The calculated kinetic constants of CXFeY followed the order of C5Fe2 (0.1024 min^−1^) > C5Fe1 (0.0908 min^−1^) > C5Fe0.5 (0.0793 min^−1^) > C3Fe1 (0.0737 min^−1^) > C3Fe0.5 (0.0689 min^−1^) > C5Fe5 (0.0682 min^−1^) > C3Fe5 (0.0658 min^−1^) > C3Fe2 (0.0607 min^−1^) > C7Fe1 (0.0365 min^−1^) > C7Fe2 (0.0336 min^−1^) > C7 (0.0286 min^−1^) > C3 (0.0228 min^−1^) > C5 (0.0212 min^−1^) > C7Fe0.5 (0.0163 min^−1^) > C7Fe5 (0.0155 min^−1^) (Figure 2d and Table S5). The calculated theoretical adsorption capacities for CXFeY are in the sequence of C5Fe5 (237.50 mg g^−1^) > C5Fe2 (223.40 mg g^−1^) > C5Fe1 (214.10 mg g^−1^) > C5Fe0.5 (212.44 mg g^−1^) > C3Fe5 (185.36 mg g^−1^) > C3Fe2 (131.53 mg g^−1^) > C3Fe1 (88.87 mg g^−1^) > C7Fe0.5 (57.11 mg g^−1^) > C3Fe0.5 (56.70 mg g^−1^) > C7Fe1 (19.40 mg g^−1^) > C7Fe2 (13.61 mg g^−1^) > C7Fe5 (9.40 mg g^−1^) > C3 (4.85 mg g^−1^) > C5 (3.25 mg g^−1^) > C7 (2.40 mg g^−1^). (Figure 2e and Table S6). The maximum adsorption capacity of CXFeY for As(III) showed a remarkable increase of 3.92–73.08 times compared with that of NC. Noticeably, Fe‐decorated C5 exhibited ultrahigh and fastest As(III) decontamination capacity among all materials. Importantly, negligible Fe leaching was observed during the reaction, with dissolved Fe concentrations ranging from 3.99 to 41.10 µg L^−1^, far below the WHO drinking–water guideline value of 300 µg L^−1^ (Figure S21). This result confirms the stable anchoring of Fe atoms within SAFeC. Overall, C5FeY demonstrated ultra–efficient As(III) removal, surpassing most reported state–of–the–art Fe–based adsorbents (Figure 2f). When normalized to Fe content, C5FeY achieved the highest normalized As(III) adsorption capacity (4520.5–39833.5 mg (g Fe)^−1^) (Figure 2g and Table S7), highlighting the exceptional utilization efficiency of atomically dispersed Fe sites.

**FIGURE 2:**
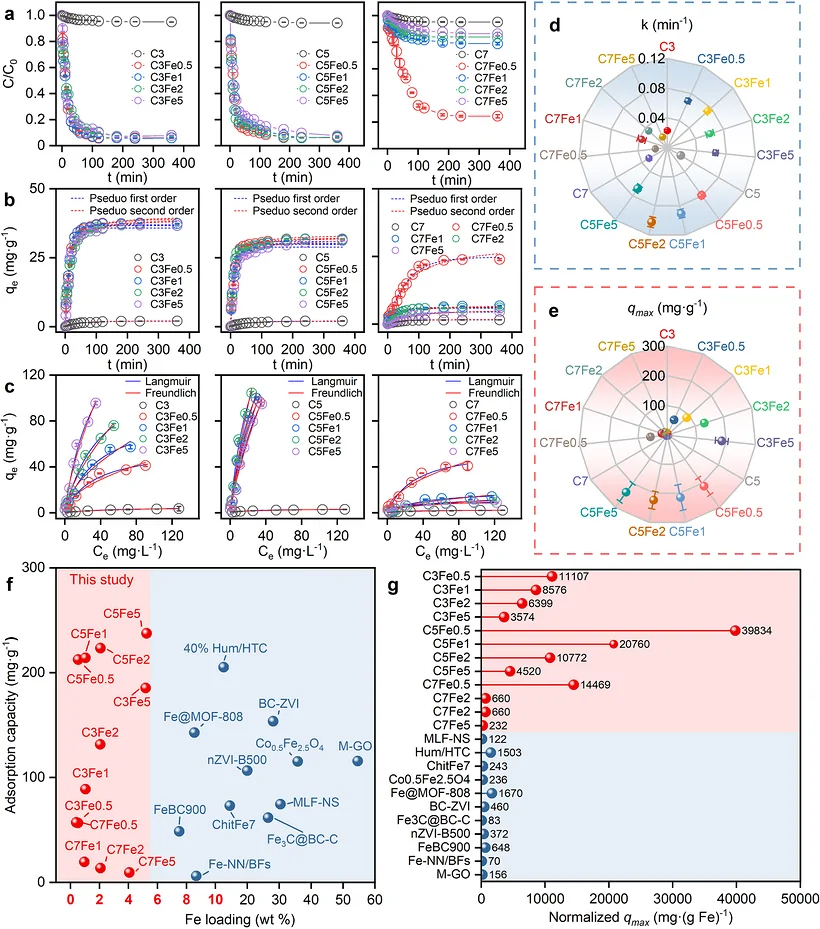
(a) Removal efficiency, (b) adsorption kinetics, and (c) adsorption isotherms of As(III) by CXFeY. Comparison of (d) adsorption rate constants and (e) maximum adsorption capacities of CXFeY for As(III). (f) Comparison of As(III) adsorption capacity and (g) nomarlized adsorption capacity with currently reported Fe‐loading adsorbent. Reaction conditions: C_0_ (As(III)) = 40 mg L^−1^ (for adsorption kinetics), C_0_ (As(III)) = 2–100 mg L^−1^ (for adsorption isotherms), CYFeX = 1 g L^−1^, background electrolyte: 0.01  NaNO_3_, initial pH = 7, T = 298.15 K.

No oxygen–driven As(III) oxidation occurred in the absence of CXFeY, and NC exhibited only limited activity (6.07%–7.72%) (Figure S22). In contrast, CXFeY, particularly C3FeY and C5FeY, rapidly oxidized As(III) to As(V) within 20 min. Speciation analysis of the residual solution showed that As(V) accounted for 40.35%–52.87% of the remaining dissolved As (Figure 3a–c). A previous study reported that biochar supported nZVI showed higher As(III) oxidation efficiency (29.7%–34.3%) compared to that of nZVI alone (27.1%), and molecular oxygen (O_2_) was activated by biochar and nZVI to generate ROS, thereby promoting As(III) oxidation [12]. In this study, the superior performance of CXFeY could be attributed to the unique coordination environment of Fe single–atoms. We further speculated that ROS generation induced by O_2_ and the synergistic interaction between Fe atoms and support played pivotal roles in As(III) oxidation, as confirmed by ROS probing and quenching experiments.

**FIGURE 3:**
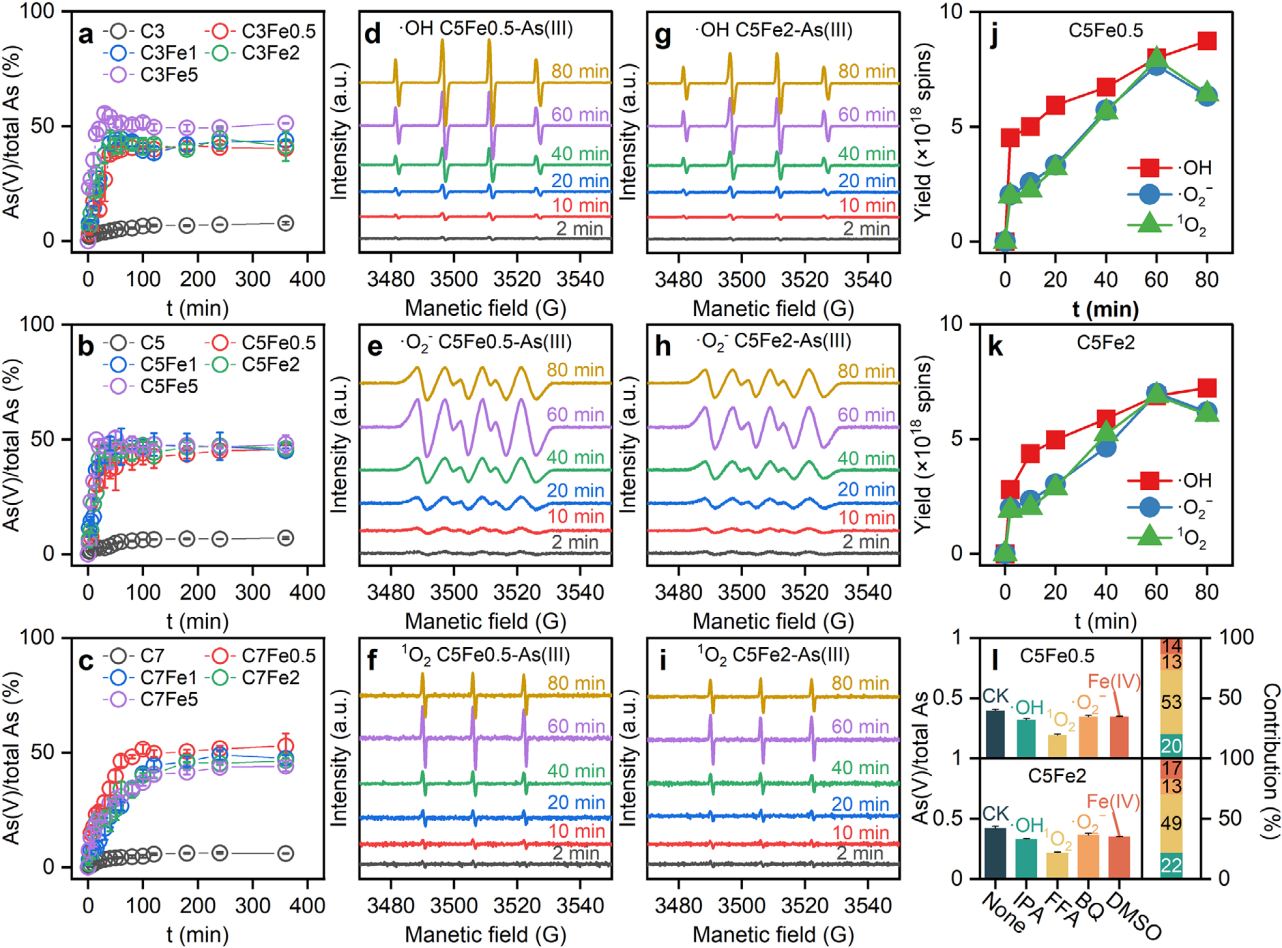
(a–c) Percentage of total As(V)/total As during reaction. (d–k) Time–resolved EPR spectra. (l) Quenching experiments in C5Fe0.5/C5Fe2‐As(III) system, and contributions of ROS for As(III) oxidation. Reaction conditions: C_0_ (As(III)) = 40 mg L^−1^, CYFeX = 1 g L^−1^, background electrolyte: 0.01 M NaNO_3_, initial pH = 7, T = 298.15 K.

### 2.3 Reaction Mechanisms

To unravel the As(III) oxidation mechanism by SAFeC, the central ROS involved in the reaction were identified by EPR and quenching experiments. No detectable ·OH, ·O_2_^−^, and ^1^O_2_ signals were observed in the As(III)–only system, confirming that dissolved O_2_ alone could not effectively oxidize As(III) or generate ROS under aerobic conditions without additional intervention (Figure S23a). In contrast, SAFeC generated distinct ·OH, ·O_2_^−^, and ^1^O_2_ signals in the presence of dissolved O_2_, and their intensities increased with increasing Fe content (Figure S23b).

During the SAFeC–As(III) reaction, the ROS signals increased progressively over time (Figure 3d–k), closely matching the trend of As(V) formation. Lower ROS accumulation in C5Fe2 compared with C5Fe0.5 indicated greater ROS consumption, consistent with its higher As(III) oxidation efficiency. Quenching experiments using specific scavengers, including IPA, BQ, FFA, and DMSO, were further conducted to clarify the contributions of different ROS (Figure 3l). The most pronounced inhibitory effect was observed with FFA, resulting in a 20% reduction in As(V) yield, indicating that ^1^O_2_ was the dominant ROS and contributed to nearly half of As(III) oxidation. The inhibition effects of IPA, BQ, and DMSO confirmed the auxiliary roles of ·OH, ·O_2_^−^, and Fe(IV), respectively. Together, EPR and quenching results demonstrate that As(III) oxidation by SAFeC was predominantly driven by non‐radical species (^1^O_2_), with radicals (·OH and ·O_2_^−^), and Fe(IV) acting synergistically.

Mechanistically, the phenolic —OH, quinone moieties, and aromatic structures in NC facilitated O_2_ activation through electron transfer, generating ROS that promoted As(III) oxidation (Reactions (1), (2), (3)) [42]. The incorporation of single‐atom Fe further enhanced this process [43]. Specifically, the robust interaction between the single–atom Fe and NC significantly enhanced the EEC of SAFeC, facilitating O_2_ activation and subsequent generation of ·OH, ·O_2_^−^, and ^1^O_2_ (Reactions (4), (5), (6), (7)), ultimately boosting As(III) oxidation (Reactions (8), (9), (10), (11), (12), (13)) [44]. The Fe atoms were stabilized as isolated sites by the NC matrix, maximizing atomic utilization and catalytic reactivity [18]. Fe(II) could undergo a single–electron transfer with O_2_ to generate ·O_2_^−^, and the resulting ·O_2_^−^ could subsequently contribute to ^1^O_2_ formation through Fe–mediated redox reactions (Reactions 14 and 15) [45, 46]. Meanwhile, NC served as an electron shuttle, mediating Fe(III)/Fe(II) cycling and maintaining the redox continuity of the SAFeC–As(III) system. Therefore, the combined EPR evidence, quenching results, and proposed O_2_ activation pathway support that ^1^O_2_ was generated through SAFeC–mediated O_2_ activation and played a dominant role in As(III) oxidation.

Further evidence for As(III) removal mechanisms was obtained through multi–scale characterization. SEM‐EDS maps demonstrated a uniform distribution of As on the SAFeC surface, substantiating its exceptional adsorption efficiency (Figure S24). After As(III) adsorption, the SAFeC surface became rougher with spherical precipitates resembling arsenate hydrate (Ca_5_(AsO_4_)_3_OH) [47]. XRD confirmed the presence of Ca_5_(AsO_4_)_3_OH (PDF#51–1501) and the decrease of Ca(OH)_2_ and CaO (Figure S25), indicating that As(III) was oxidized to As(V), followed by precipitation as Ca_5_(AsO_4_)_3_OH (Reactions 16 and 17). FTIR spectra revealed that the —OH peak at 3643 cm^−1^ redshifted and broadened after adsorption (Figure S26), confirming Ca_5_(AsO_4_)_3_OH formation and potential As —OH complexation [48]. The enhanced peaks at 870 and 603 cm^−1^ corresponded to As —O stretching vibrations, indicating the formation of hydrogen‐bonding between As—O and Fe—OH sites (870 cm^−1^) and the generation of As —O —Fe monodentate mononuclear complexes (603 cm^−1^) [49, 50]. These findings demonstrated that As(III) removal involved not only precipitation but also surface complexation and hydrogen bonding.

XPS analysis provided further insights into surface interactions. In As 2p spectra, a new peak near 1326 eV, confirmed successful As adsorption (Figure S27). Deconvolution revealed the coexistence of As(III) (28.66%–63.73%) and As(V) (36.27%–71.34%), indicating both the direct adsorption of As(III) and the subsequent adsorption of its oxidized product As(V). Fe 2p spectra indicated partial oxidation of Fe(II) to Fe(III) (0.48%–46.54%) (Figure S28), highlighting Fe(II) as the active species in O_2_ activation. O 1s spectra showed binding energy shifts of O = C, suggesting electron gain and semiquinone radical formation that facilitated As(III) oxidation. Decreases in O —C, O—Fe, and Fe—N contents after adsorption (Figures S29 and S30) supported the formation of As—O and As—O—Fe complexes, consistent with FTIR results.

The As K–edge XANES spectra confirmed partial oxidation of As(III) to As(V) in both C5Fe0.5 and C5Fe2 (Figure 4a). LCF results demonstrated that As(III) predominated in C5Fe0.5 (74.2%), whereas As(V) prevailed in C5Fe2 (56.9%) (Figure 4b), consistent with the XPS results. As K–edge FT–EXAFS spectra elucidated the coordination environments (Figure 4c and Table S8). In comparison with C5Fe2, the As—O interatomic distance (*R*) in C5Fe0.5 was longer, which was attributed to the higher proportion of As(III) in C5Fe0.5 [51]. The *R*_As—O_ for C5Fe0.5 was 1.81 Å, which closely resembled the *R*_As—O_ (1.80 Å) observed in previous studies of arsenite (AsO_3_) pyramid coordination with O‐containing functional groups in peat [52]. This suggested that the O–containing functional groups (e.g., —OH and —COOH) in C5Fe0.5 formed As—O complexes by nucleophilic attack to partially positively charged As atom (Reactions 18 and 19) [53]. The *R*_As—O_ for C5Fe2 was 1.76 Å, which was close to the typical *R*_As —O_ (1.72 Å) for the arsenate (AsO_4_) tetrahedron [54]. A second shell peak at 2.72 Å in the C5Fe2–As EXAFS spectrum corresponded to As—Fe scattering (*R*_As—Fe_ of 2.92 Å), indicating inner–sphere complex formation between As and Fe [55]. The coordination structure of As in C5Fe2 closely resembled that of FeAsO_4_ (Figure 4d). A previous study has reported that FeAsO_4_ consisted of isolated FeO_6_ octahedra and four distinct AsO_4_ tetrahedra that were linked through corner–sharing O atoms [56]. In contrast, the coordination number of As—Fe in C5Fe2 was 1.2, and Fe existed as isolated Fe atoms, which indicated that AsO_4_ did not form bidentate binuclear complexes with Fe in C5Fe2 [57]. Considering the As(III/V) fraction in C5Fe2, we speculated that the AsO_3_ pyramid or AsO_4_ tetrahedra (oxidized As(III)) interacted with the O atoms located outside the FeN_4_ plane in C5Fe2 (where Fe atoms adopted the FeN_4_O configuration), forming Fe—O—As monodentate mononuclear complexes through corner–sharing (Reactions 20 and 21). Based on the above results, a schematic illustration of the proposed adsorption–oxidation mechanisms of As(III) by SAFeC is shown in Figure 4e.

(1) $$\mathrm{quinoid}\;\left(C=O\right)+e^{-}\;\to \mathrm{semiquinone}\;\mathrm{radical}\;\left(C=O\cdot^{-}\right)$$

(2) $$\begin{array}{cc} & \mathrm{semiquinone}\;\mathrm{radical}\;\left(C=O\cdot^{-}\right)+\mathrm{As}\left(\mathrm{III}\right)\\ & \to \mathrm{As}\left(\mathrm{IV}\right)+\;\mathrm{phenolic}-\mathrm{OH} \end{array}$$

(3) $$\mathrm{As}\left(\mathrm{IV}\right)+O_{2}\to \mathrm{As}\left(V\right)+\cdot {O_{2}}^{-}$$

(4) $$O_{2}+e^{-}\to \cdot {O_{2}}^{-}$$

(5)

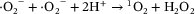

(6) $$H_{2}O_{2}+\cdot {O_{2}}^{-}\to O_{2\;}+\cdot \mathrm{OH}+H_{2}O$$

(7) $$\cdot {O_{2}}^{-}+\cdot \mathrm{OH}\;\to^{1}O_{2}+{\mathrm{OH}}^{-}$$

(8) $$\mathrm{As}\left(\mathrm{III}\right)+\cdot {O_{2}}^{-}+H^{+}\to \mathrm{As}\left(\mathrm{IV}\right)+{\mathrm{HO}}_{2}^{-}$$

(9) $$\mathrm{As}\left(\mathrm{IV}\right)+\cdot {O_{2}}^{-}+H^{+}\to \mathrm{As}\left(V\right)+{\mathrm{HO}}_{2}^{-}$$

(10) $$\mathrm{As}\left(\mathrm{III}\right)+\cdot \mathrm{OH}\to \mathrm{As}\left(\mathrm{IV}\right)+OH^{-}$$

(11) $$\mathrm{As}\left(\mathrm{IV}\right)+\cdot \mathrm{OH}\to \mathrm{As}\left(V\right)+OH^{-}$$

(12) $$\mathrm{As}\left(\mathrm{III}\right){+}^{1}O_{2}+H^{+}\to \mathrm{As}\left(\mathrm{IV}\right)+H_{2}O$$

(13) $$\mathrm{As}\left(\mathrm{IV}\right){+}^{1}O_{2}+H^{+}\to \mathrm{As}\left(V\right)+H_{2}O$$

(14) $$\mathrm{Fe}\left(\mathrm{II}\right)+O_{2}\to \mathrm{Fe}\left(\mathrm{III}\right)+\cdot {O_{2}}^{-}$$

(15) $$\mathrm{Fe}\left(\mathrm{III}\right)+\cdot {O_{2}}^{-}\to \mathrm{Fe}\left(\mathrm{II}\right){+}^{1}O_{2}$$

(16) $$5{\mathrm{Ca}}^{2+}+3{\mathrm{HAsO}}_{4}^{2-}+4{\mathrm{OH}}^{-}\to {\mathrm{Ca}}_{5}{\left({\mathrm{AsO}}_{4}\right)}_{3}\mathrm{OH}↓+3H_{2}O$$

(17) $$5{\mathrm{Ca}}^{2+}+3{\mathrm{AsO}}_{4}^{3-}+O\$H^{-}\to {\mathrm{Ca}}_{5}{\left({\mathrm{AsO}}_{4}\right)}_{3}\mathrm{OH}↓$$

(18) $$R-\mathrm{COOH}+\mathrm{As}{\left(\mathrm{OH}\right)}_{3}\to R-\mathrm{COO}-\mathrm{As}{\left(\mathrm{OH}\right)}_{2}+H_{2}O$$

(19) $$R-\mathrm{OH}+\mathrm{As}{\left(\mathrm{OH}\right)}_{3}\to R-O-\mathrm{As}{\left(\mathrm{OH}\right)}_{2}+H_{2}O$$

(20) $$N_{4}\mathrm{Fe}-\mathrm{OH}+\mathrm{As}{\left(\mathrm{OH}\right)}_{3}\;\to \;N_{4}\mathrm{Fe}-O-\mathrm{As}{\left(\mathrm{OH}\right)}_{2}+H_{2}O$$

(21) $$N_{4}\mathrm{Fe}-\mathrm{OH}+{\left(\mathrm{OH}\right)}_{3}\mathrm{AsO}\to N_{4}\mathrm{Fe}-O-\mathrm{As}{\left(\mathrm{OH}\right)}_{2}O+H_{2}O$$

**FIGURE 4:**
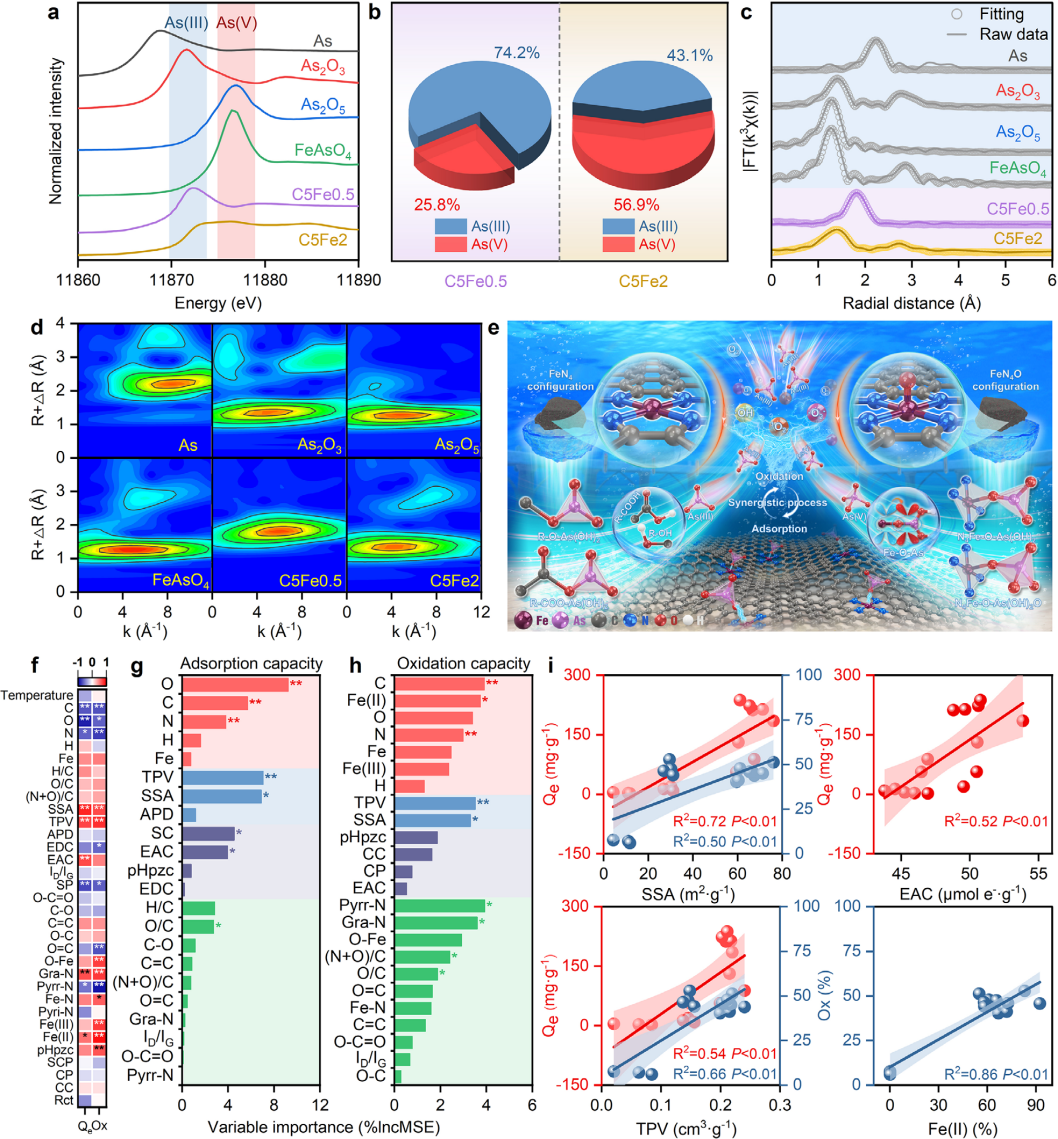
(a) Normalized As K–edge XANES spectra of references and SAFeC. (b) As Speciation of in SAFeC based on LCF analysis. (c) As K–edge FT–EXAFS spectra and EXAFS fitting of reference samples and SAFeC. (d) WT analysis. (e) Proposed mechanism of As(III) adsorption–oxidation by SAFeC. (f) Correlation matrix between CXFeY characteristics and As(III) adsorption capacity (Q_e_) or oxidation capacity (O_X_). Random Forest mean predictor importance of CXFeY characteristics on (g) Q_e_ and (h) O_X_. (i) Linear regression relationships between key CXFeY characteristics and Q_e_/O_X_. ^*^ *p* < 0.05; ^**^ *p* < 0.01.

### 2.4 Quantitative Structure–Activity Relationship

A quantitative structure–activity relationship analysis was established to correlate intrinsic properties of SAFeC with its As(III) adsorption–oxidation performance. Correlation analysis showed that As(III) adsorption capacity was associated with SSA, TPV, EAC, graphitic—N, and Fe(II) content, while As(III) oxidation correlated positively with SSA, TPV, O—Fe, graphitic—N, Fe—N, Fe(II), and Fe(III) (*p* < 0.05) (Figure 4f). The random forest model identified the overall importance of material features as follows: for adsorption, elemental composition > pore characteristics > chemical structural features > electrochemical properties; and for oxidation, chemical structural features > elemental composition > pore structure > electrochemical properties. Among these descriptors (Figure 4g,h), SSA (R^2^ = 0.72) and TPV (R^2^ = 0.54) exhibited stronger correlation with As(III) adsorption capacity than EAC (R^2^ = 0.52), while Fe(II) content (R^2^ = 0.86) showed the strongest correlation with As(III) oxidation capacity (Figure 4i). These results indicated that pore characteristics (SSA and TPV) dominated As(III) adsorption, whereas Fe(II) content played a critical role in As(III) oxidation.

Enhancing the pore structure effectively prevented Fe aggregation, ensured uniform dispersion of single–atom Fe, and increased the surface free energy of Fe atoms, enabling each Fe atom to actively participate in the As(III) adsorption and oxidation process [58]. The enlarged SSA and TPV facilitated As(III) diffusion and O_2_ penetration, optimized the electron and ROS transfer pathways, and prolonged redox intermediate lifetimes, thereby amplifying adsorption and O_2_–induced oxidation of As(III) [59, 60, 61]. Increased EAC strengthened the electrostatic adsorption of As(III) onto SAFeC [62], and the elevated Fe(II) content facilitated O_2_ activation and ROS generation, accelerating As(III) oxidation [63].

### 2.5 Theoretical Calculation

The origin of the exceptional As(III) removal performance of FeSAC was further comprehended using theoretical calculations, focusing on electronic structures, reaction energy, and binding pathways. The electrostatic potential distribution on the van der Waals surface revealed that Fe atom incorporation induced significant electron density redistribution in the pristine NC (Figure 5a). Specifically, the introduction of Fe decreased the electron density in the electron–rich regions around the pyridinic—N atoms in the NC structure, facilitating coordination between the positively charged Fe centers and the electron–donating N atoms. This coordination transformed the originally electron–rich pyridinic sites into electron–deficient regions centered on Fe, thus enhancing their affinity for electron–rich As(III). In C5Fe2, electronic interactions between Fe and O atoms induced the localization of Fe d–electrons, causing them to deviate from the Fe atoms toward the axial O atom. This suggests that the axial O coordination in FeN_4_O could further modulate the electronic structure of the Fe center, making it more favorable for As(III) adsorption than FeN_4_. Molecular orbital calculations indicated that the pristine NC exhibited a wide LUMO–HOMO gap (E_gap_ = 1.41 eV), whereas Fe single–atom incorporation introduced Fe—N hybrid orbitals that narrowed the E_gap_ (0.91–1.13 eV for SAFeC) (Figure 5b), thereby enhancing electron transfer and chemical reactivity [64].

**FIGURE 5:**
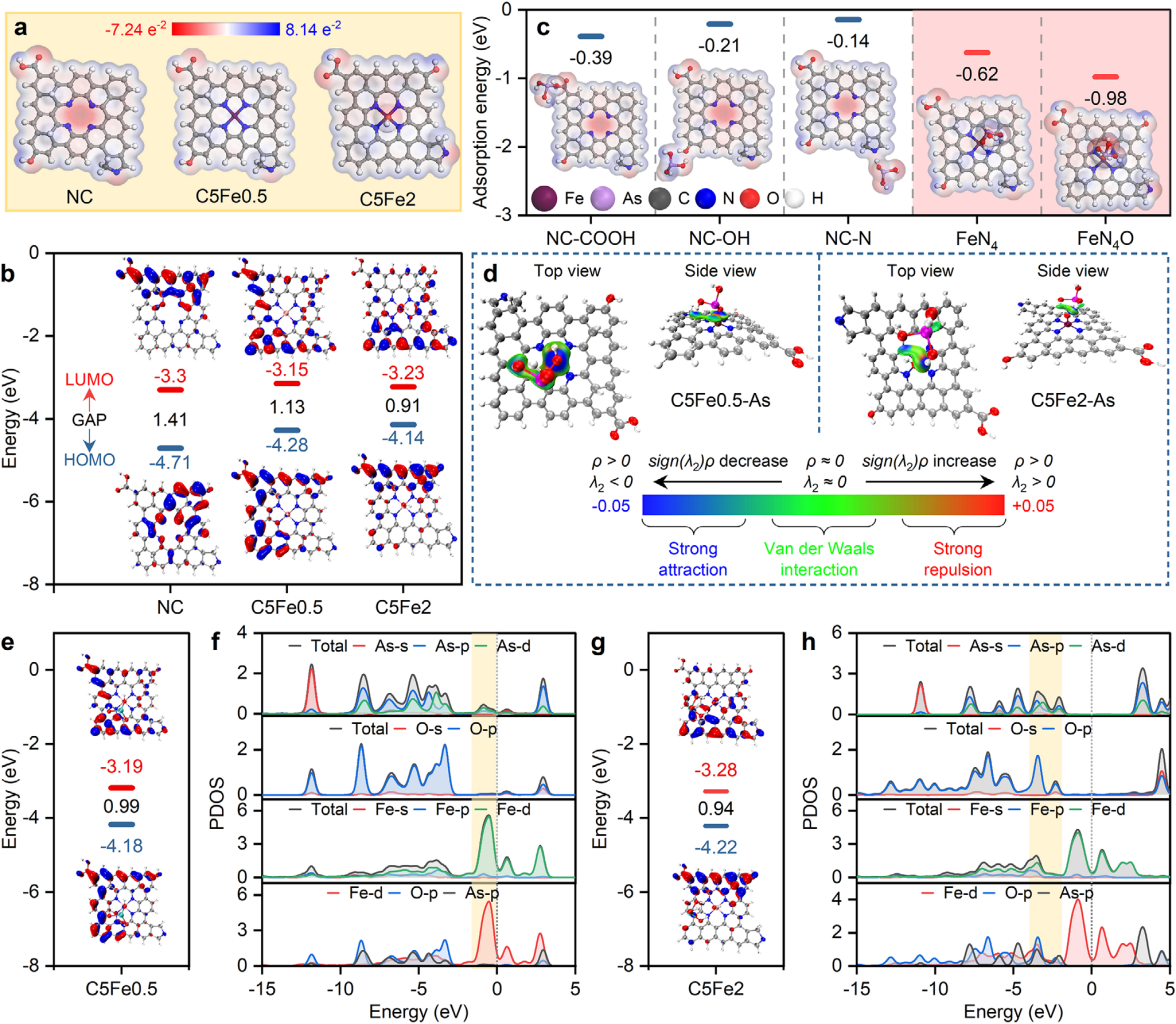
(a) Electrostatic potential plots of SAFeC. (b) HOMO and LUMO of SAFeC. (c) Adsorption energy of SAFeC with different configurations for As(III). (d) IGM scatter diagrams of SAFeC for As(III) adsorption. (e,g) HOMO and LUMO of SAFeC after As(III) adsorption. (f,h) DOS and PDOS plots of SAFeC after As(III) adsorption.

The adsorption energies of As(III) at various reaction sites in SAFeC are depicted in Figure 5c. Compared to NC, the adsorption energies of As(III) at various binding sites in SAFeC were notably enhanced. The calculated As(III) binding affinity followed the order: FeN_4_O (−0.98 eV) > FeN_4_ (−0.62 eV) > NC—COOH (−0.39 eV) > NC—OH (−0.21 eV) > NC—pyrrolic—N (−0.14 eV), confirming that Fe sites were the most favorable binding centers. This result indicated that atomically dispersed Fe centers contribute more effectively to As(III) adsorption than the intrinsic O/N–containing groups on NC, while axial O coordination further strengthened the adsorption ability of FeN_4_O. IGM analysis indicated intense δginter signals at sign(λ2)ρ ≈ –0.05 a.u., indicative of hydrogen bonding (Figure 5d). The O atom in As(OH)_3_ formed strong hydrogen bonds with Fe in FeN_4_ configuration, while in FeN_4_O, the O atom situated outside the FeN_4_ plane formed strong hydrogen–bonding with the H atom in As(OH)_3_. The green–colored areas indicated that As(OH)_3_ was also inclined to interact with the FeN_4_ plane through van der Waals interactions.

Upon As(III) adsorption, the LUMO‐HOMO gap of the SAFeC–As(III) complexes further decreased (Figure 5e,g), suggesting charge redistribution and stable SAFeC–As(III) complex formation. PDOS analysis revealed significant orbital overlap among As 2p, O 2p, and Fe 3d below the Fermi level, especially in C5Fe2 (Figure 5f,h), indicating electron sharing among Fe, As, and O [65]. Compared with C5Fe0.5, the C5Fe2–As(III) system exhibited a more pronounced PDOS overlap, with Fe—As hybrid orbitals shifted to a lower energy range (−13.59 to 0 eV versus −9.37 to 0 eV), indicating the formation of more stable Fe—O—As bonds [66]. Therefore, the enhanced As(III) adsorption on FeN_4_O could be attributed to axial O–induced electronic modulation of Fe 3d orbitals, which strengthened Fe—O—As orbital coupling and promoted interfacial charge transfer. This aligned with experimental results showing that C5Fe2 exhibited higher As(III) adsorption capacity. Overall, the DFT results corroborated the XPS and EXAFS findings, emphasizing that atomically dispersed Fe centers acted as the critical active sites that strengthened As(III) removal by SAFeC.

### 2.6 Environmental Robustness

To evaluate the environmental robustness of SAFeC, the effects of solution pH, water matrices, co–existing anions, and ionic strength on As(III) removal were systematically examined. At an initial As(III) concentration of 1 mg L^−1^, C3FeY and C5FeY exhibited excellent As(III) removal performance over a wide pH range of 3–11, with removal efficiencies ranging from 85.86% to 96.69% (Figure S32). As the pH increased from 3 to 7, the As(III) removal efficiency of C3FeY and C5FeY gradually increased, reaching a maximum of 94.91%–96.69%. At higher pH values (7–11), the deprotonated As(III) species, such as H_2_AsO_3_^−^ and HAsO_3_^2−^, may experience stronger electrostatic repulsion from the SAFeC surface and compete with OH^−^ for adsorption sites, resulting in a slight decrease in As(III) removal efficiency [26]. Nevertheless, C5FeY still maintained approximately 90% As(III) removal efficiency at pH 9 and 11. Attractively, C5FeY showed excellent practical applicability in real waters, achieving 96.57%–98.02% removal in river water, tap water, and drinking water (Figure S33). Coexisting anions at concentrations up to 1.0 mM had negligible effects on As(III) removal by C5FeY (Figure S34). In particular, phosphate showed only a limited inhibitory effect, in sharp contrast to many Fe–based adsorbents, such as Fe–biochar fibers and Fe–Mn binary oxides, which typically suffer from a >50% decrease in As(III) removal efficiency in the presence of phosphate due to its chemical similarity to As species [67, 68]. The superior tolerance of C5FeY toward competing anions highlights its strong anti‐interference capacity. Similarly, increasing ionic strength exerted little influence on As(III) removal, indicating that As(III) removal by C5FeY mainly proceeded through inner–sphere surface complexation [38].

To further assess the practical applicability of SAFeC under environmentally relevant As(III) levels, C5Fe0.5 and C5Fe2 were selected as representative samples for additional tests at a lower initial As(III) concentration of 100 µg L^−1^ (Figure 6). Both samples maintained high As(III) removal efficiencies under various conditions, including a broad pH range of 3–11, co‐existing anions (NO_3_^−^, CO_3_^2−^, SO_4_^2−^, SiO_3_^2−^, and PO_4_^3−^) at 0.1–1.0 mm, the presence of humic acid (HA), and real water matrices, including river water, drinking water, and tap water (Figure 6a–e). Importantly, the residual As concentrations after treatment were consistently below the regulatory limit for As in drinking water of 10 µg L^−1^, providing direct evidence for the practical applicability of SAFeC in drinking water treatment. In addition, the reusability tests showed that SAFeC retained over 90% As(III) removal efficiency after six regeneration cycles, accompanied by low Fe leaching, demonstrating its good operational stability and structural robustness (Figure 6f). From a broader environmental perspective, the efficient removal of As(III) from contaminated water is highly relevant to global sustainability goals, particularly United Nations Sustainable Development Goal 6, which emphasizes access to safe drinking water and sustainable water management. The SAFeC system developed in this study can reduce environmentally relevant As(III) concentrations to below the WHO drinking–water guideline value of 10 µg L^−1^ under various water chemistry conditions, while maintaining good reusability and low Fe leaching during repeated treatment cycles. In addition, the use of atomically dispersed Fe sites improves the utilization efficiency of active metal centers, potentially lowering material demand and secondary environmental burdens. These characteristics highlight the potential of SAFeC as a sustainable and practical strategy for arsenic risk mitigation and water purification.

**FIGURE 6:**
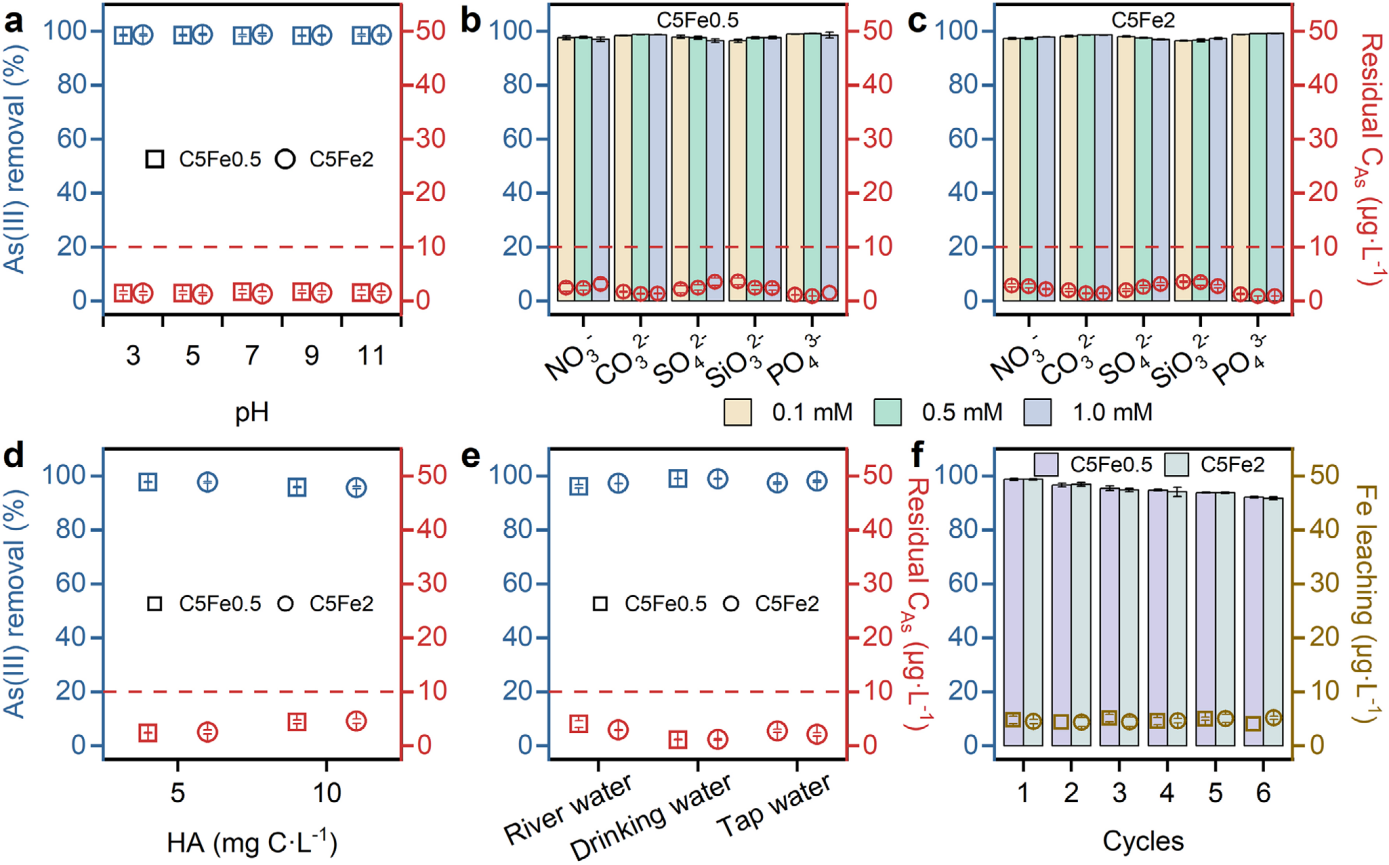
Effect of (a) initial pH, (b,c) co‐existing anions and ionic strength, (d) humic acid, and (e) water matrix on As(III) removal by SAFeC. (f) Reusability tests and Fe leaching during regeneration cycles. Reaction conditions: *C*_0_ (As(III)) = 100 µg L^−1^, SAFeC = 1 g L^−1^, T = 298.15 K, eluting agent = 1  m NaOH.

These results demonstrate the good environmental robustness of SAFeC under the tested conditions. Nevertheless, long–term stability under realistic and dynamic water treatment scenarios warrants further investigation. Future studies should systematically evaluate the aging behavior, leaching/desorption performance, and structural evolution of spent SAFeC during prolonged exposure to variable pH, temperature, ionic strength, competing ions, natural organic matter, and real water matrices. Monitoring As release, Fe leaching, As speciation, and surface structural changes will provide deeper insight into the stability of As immobilization and Fe active sites, thereby supporting a more comprehensive assessment of the durability and field applicability of SAFeC.

## 3 Conclusions

In this study, a single–atom Fe–based remediation material (SAFeC) was rationally designed through precise optimization of support structure and Fe coordination environment. The atomic dispersion of Fe atoms and the well–defined Fe—N coordination structure synergistically enhanced the electrochemical activity, surface functionality, and pore structure of SAFeC, resulting in outstanding As(III) adsorption–oxidation capacity, rapid kinetics, and remarkable stability under diverse aqueous conditions. Comprehensive spectroscopic analyses and theoretical calculations revealed that As(III) removal was predominantly governed by Fe—O—As monodentate inner–sphere complexation, precipitation, hydrogen bonding, and electrostatic attraction, while the atomically dispersed FeN_4_/FeN_4_O sites effectively activated O_2_ to selectively generate ^1^O_2_, thus driving efficient As(III) oxidation without external oxidants. Importantly, this work demonstrates a promising “waste–to–remedy” strategy that valorizes N–rich kitchen waste (crayfish shells) into a high–performance SAFeC. This sustainable synthesis approach not only minimizes biomass waste but also offers a cost–effective and scalable route for producing single–atom–doped functional materials. In future applications, the facile synthesis and robust adsorption‐oxidation performance of SAFeC render it promising for real–water applications, including industrial effluents and As‐contaminated groundwater. Continued optimization of synthesis parameters, reactor design, and regeneration techniques could further enhance its practicality and accelerate the translation of this waste–derived single–atom technology from laboratory research to field–scale implementation. Overall, this study represents a significant breakthrough in sustainable environmental remediation, offering both mechanistic insights and a realistic pathway toward the scalable treatment of As–contaminated water.

## 4 Experimental Section

### 4.1 Synthesis of SAFeC

The crayfish shell (CS) was obtained from post–consumption food residues of local restaurants in Jiangsu, China, representing a typical source of kitchen waste. The collected CS was washed with ultrapure water to remove impurities, dried at 90°C, and then ground through a 100–mesh sieve. Elemental analysis revealed that CS contained 23.4%, 3.2%, 31.2%, 4.2%, and 21.9% of C, H, O, N, and Ca. XRD and FTIR results exhibited characteristic peaks of typical CaCO_3_ and chitin in CS (Figure S1). The biomass powder was pyrolyzed at 300°C, 500°C, and 700°C under an N_2_ atmosphere for 2 h at a heating rate of 10°C·min^−1^ in a tube furnace. The product was collected after cooling to room temperature. Then, the obtained powder was washed repeatedly with ultrapure water, and dried for later use. Based on the pyrolysis temperature, the N–rich carbon–based support (NC) was labeled as C3, C5, and C7, respectively. Subsequently, the NC and FeCl_3_·6H_2_O were mixed in aqueous solution at different mass ratios of Fe and carrier (Fe: carrier = 0.5%, 1%, 2%, and 5%), and stirred at 25°C for 24 h. The mixed solid was filtered and dried at 40°C. The dried solid was secondary carbonized in a tube furnace (850°C, 10°C·min^−1^, 1 h, N_2_ atmosphere). Finally, the single–atom Fe decorated N–rich porous biochar was obtained, which was named as CXFeY (the variables X and Y represented the carrier type and the Fe doping amount, respectively).

### 4.2 Experimental and Analytical Methods

A kinetics experiment was performed to study As(III) adsorption and oxidation processes by CXFeY under aerobic conditions. The reactions were conducted under ambient atmospheric conditions, with continuous stirring at 200 rpm to maintain dissolved oxygen saturation at 298.15 K. The initial As(III) concentration was 40 mg L^−1^ and the CXFeY dosage was 1 g L^−1^. The working solution contained 0.01 M NaNO_3_ as the background electrolyte and the initial solution pH was adjusted to 7 using HNO_3_/NaOH. The samples were withdrawn at specific times (5–360 min) and filtered through 0.22–µm filter for subsequent analysis. After the reaction, the solid was collected, washed with ultrapure water, and freeze–dried for later characterization. Isothermal adsorption experiments were performed to assess the maximum adsorption capacities of CXFeY for As(III). The initial concentration of As(III) in the isothermal experiment ranged from 2 to 100 mg·L^−1^ and the reaction time was 180 min. All other reaction conditions were consistent with those in the kinetics experiments.

The effects of initial pH (3, 5, 7, 9, and 11), coexisting ions (NO_3_^−^, CO_3_^2−^, SO_4_^2−^, SiO_3_^2−^, and PO_4_^3−^), ionic strength (0.1, 0.5, and 1.0 mM), and water matrix (river water, tap water, and drinking water) on As(III) adsorption and cyclic experiments were systematically investigated to explore the practical application potential of SAFeC. In the cyclic experiments, the As–loaded SAFeC was regenerated using 1 m NaOH as the eluent for 6 h. The initial As(III) concentration and CXFeY dosage in these experiments were 1000 µg L^−1^ and 1 g L^−1^, respectively. All experiments were repeated in triplicate.

The reactive oxygen species (ROS) generated in the As(III) oxidation process were examined using an electron paramagnetic resonance spectrometer (EPR, Bruker E500). The DMPO and TEMP were used as spin–trapping agents to capture ·OH/·O_2_^−^ and ^1^O_2_, respectively. The ROS spin concentration (SP) was quantified by comparing the signal peak areas to the standard 2,2–diphenyl–1–picrylhydrazyl [42]. The quenching experiments were conducted by adding isopropanol (IPA), p–benzoquinone (BQ), furfuryl alcohol (FFA), and dimethyl sulfoxide (DMSO) to selectively quench ·OH, ·O_2_^−^, ^1^O_2_, and Fe(IV), respectively [69, 70]. These quenchers were selected for their high selectivity and near diffusion‐controlled reaction rate constants (typically 10^8^–10^9^ m ^−1^·s^−1^) with the corresponding ROS. Total As and As species (As(III) and As(V)) concentrations in the solution were determined using an atomic fluorescence spectrophotometer (AFS, Haiguang 8520) and high–performance liquid chromatography combined with AFS (HPLC–AFS, Haiguang 9770), respectively. The total Fe concentration in the filtrate was quantified using inductively coupled plasma mass spectrometry (ICP–MS, iCAP Q, USA).

### 4.3 Characterizations

The content of non–metallic elements (C, O, N, H and S) and metallic elements (Fe, Mn, K, Na, Ca, Mg and Mn) in SAFeC were examined by an elemental analyzer (Euro Vector EA3000) and ICP–MS, respectively. The microstructure and elemental distribution of SAFeC were characterized using a scanning electron microscope (SEM, Gemini SEM 500), high–resolution transmission electron microscope (HRTEM, FEI Tecnai G2 F200) and aberration–corrected high–angle annular dark‐field scanning transmission electron microscopy (AC–HAADF–STEM, FEI Titan G2 60–300) equipped with energy dispersive spectroscopy (EDS). The specific surface area and pore size of SAFeC was determined using N_2_ adsorption–desorption isotherms (ASAP2460) and analyzed by Brunauer–Emmett–Teller (BET) and Barrett–Joyner–Halenda (BJH) methods. The crystalline structure of SAFeC was tested using an X–ray diffractometer (XRD, X'Pert Pro MPD). The surface functional groups of SAFeC were tested by Fourier transform infrared spectroscopy (FTIR, Nicolet iS10) and further explored using two–dimensional correlation spectroscopic (2D–COS) analysis. The defect and graphitization degrees of SAFeC were detected by Raman spectroscopy (RM2000). The surface defects and free radicals of SAFeC were determined using an EPR spectrometer (Bruker E500). The valence state and chemical form of surface elements on the SAFeC surface were investigated using X–ray photoelectron spectroscopy (XPS, ESCALAB 250Xi). The zeta potential of SAFeC was analyzed using a Zetasizer (Nano‐ZS90). The electrochemical measurements including electron–accepting capacity (EAC), electron–donating capacity (EDC), electron exchange capacity (EEC), cyclic voltammetry (CV) curves, Tafel curves, and electrochemical impedance spectroscopy (EIS) were measured using a CHI660D electrochemical workstation with a standard three–electrode system (reference electrode: Ag/AgCl, counter electrode: platinum plate, working electrode: glassy carbon). The speciation and coordination environment of Fe and As in SAFeC was unveiled using X–ray absorption spectroscopy (XAS), which was carried out at the Shanghai Synchrotron Radiation Facility (SSRF).

### 4.4 Theoretical Calculation

All theoretical calculations, including electrostatic potential, reaction energy, weak interaction, and molecular orbital analysis were implemented in Gaussian.

### 4.5 Statistical Analysis

All statistical analysis were performed in R software (v.4.4.1). One–way analysis of variance (ANOVA) with least significant difference (LSD) test using the “agricolae” package was conducted to analyze the significant differences among treatments. The relationship between the characteristics and adsorption–oxidation capacity of SAFeC for As was explored using correlation analysis and regression analysis in the “corrplot” and “ggpmisc” packages. The key physicochemical parameters that drove the As adsorption–oxidation capacity were identified using random forest analysis in the “randomForest” package.

## Author Contributions

**Tao Sun**: conceptualization, data curation, methodology, investigation, writing – original draft. **Chao Wang**: investigation, methodology. **Shihang Wu**: writing – review & editing, methodology. **Xiaojia Zhou**: investigation, writing – review & editing. **Penggang Pei**: conceptualization, methodology, investigation, writing – review & editing. **Huijuan Yu**: methodology, software. **Yun Zhang**: methodology, investigation. **Qingqing Huang**: writing – review & editing. **Yuebing Sun**: writing – review & editing, conceptualization, project administration, supervision.

## Funding

This work was supported by Innovation Program of Chinese Academy of Agricultural Sciences (CAAS‐CSGLCA‐202302), the National Natural Science Foundation of China (42307056), National Key Research and Development Program of China (2024YFD1701100), and Tianjin Natural Science Foundation Project (25JCZDJC00370).

## Conflicts of Interest

The authors declare no conflict of interest.

## Supporting information




**Supporting File**: advs76858‐sup‐0001‐SuppMat.docx.

## Data Availability

The data that support the findings of this study are available from the corresponding author upon request.
