# Supplementary material for: Single–Atom Iron Sites Enable Enhanced Arsenic Removal From Water Through Synergistic Adsorption–Oxidation
Source: Adv Sci (Weinh). 2026 Jul 29;13(59):e76858. doi: 10.1002/advs.76858 (PMC13418736; doi:10.1002/advs.76858)
Supplement: Supplementary file 1 [file ADVS-13-e76858-s001.docx]

Supplementary Information

Single-Atom Iron Sites Enable Enhanced Arsenic Removal from Water through Synergistic Adsorption-Oxidation

*Tao Sun, Chao Wang, Shihang Wu, Xiaojia Zhou, Penggang Pei, Huijuan Yu, Yun Zhang, Qingqing Huang, Yuebing Sun*^*^

Supporting information for the article includes Supplementary Text, Supplementary Figures (Figures S1-S34), Supplementary Tables (Tables S1-S8).

# Supplementary Text

Chemicals

Sodium arsenite (NaAsO_2_, ≥ 90%), iron chloride hexahydrate (FeCl_3_·6H_2_O, ≥ 99%), sodium nitrate (NaNO_3_, ≥ 99%), sodium carbonate (Na_2_CO_3_, ≥ 99%), sodium sulfate (Na_2_SO_4_, ≥ 99%), sodium metasilicate (Na_2_SiO_3_·9H_2_O, ≥ 98%), trisodium phosphate (Na_3_PO_4_·12H_2_O, ≥ 98%), Sodium hydroxide (NaOH ≥ 97%), nitric acid (HNO_3_), 5,5-dimethyl-1-pyrroline-N-oxide (DMPO), 2,2,6,6-tetramethyl-4-piperidinyl (TEMP), isopropanol (IPA), p-benzoquinone (BQ), furfuryl alcohol (FFA) and dimethyl sulfoxide (DMSO) were purchased from Sigma-Aldrich. All chemical used in this study were analytical grade. All solutions were prepared with Milli-Q water (18.2 MΩ).

Electrochemical Measurements

EDC and EAC were determined using the amperometric i-t method. For EDC measurement, the applied redox potential was set at 0.61 V. Electrolysis was performed in an electrolytic cell containing 2.5 mL phosphate buffer (pH 7) and 25 mL 0.2 M KCl. The working electrode was inserted into the cell and connected to the workstation, and measurements were conducted in the “Amperometric i-t curve” mode (total duration 4000 s). After a 600 s baseline stabilization, 1 mL of mediator 2,2'-azino-bis(3-ethylbenzothiazoline-6-sulfonic acid) (4 g·L^-1^) was added. Once the signal stabilized (~600 s), the target solution was introduced. For EAC measurement, the redox potential was set at -0.49 V, with diquat dibromide hydrate (2  g·L^-1^) as the mediator. EDC/EAC = *A_p_* / (*e* × *m* × *N_a_*), where *A_p_* is the integrated charge from the oxidation or reduction curve (C), *e* is the elementary charge (1.6 × 10^-19^ C), *m* is sample mass in system, and *N_a_* is Avogadro’s constant (6.02×10^23^ mol^-1^). CV measurements were performed in 0.5 M Na_2_SO_4_ solution, with a scan rate of 10 mV·s^-1^ and a potential range of 0-1 V. EIS measurements were conducted in a solution containing 1 mM K_3_Fe(CN)_6_, 1 mM K_4_Fe(CN)_6_, and 0.1 M KCl after deoxygenation. The frequency range was 0.1 Hz to 10^6^ Hz, with an AC amplitude of 5 mV.

X-ray absorption spectroscopy (XAS) measurement and data analysis

The Fe and As K-edge X-ray absorption near edge structure (XANES) and extended X-ray absorption fine structure (EXAFS) spectra was collected at the BL14W1 beamlines of Shanghai Synchrotron Radiation Facility (SSRF), Shanghai Institute of Applied Physics (SINAP). A Si (III) double-crystal monochromator was used to filter the X-ray beam. The Fe foil, FeO, Fe_2_O_3_, FePc, As Foil, As_2_O_3_, As_2_O_5_ and FeAsO_4_ was used as references and all samples were measured under transmission mode using a Lytle detector at room temperature. The energy of beam was calibrated with Fe foil (7112 eV) and Au foil (11919 eV) for Fe and As measurement, respectively.

The EXAFS oscillations *χ*(*k*) were extracted and analysed using the Demeter software. The acquired EXAFS data were processed according to the standard procedures using the ATHENA module implemented in the IFEFFIT software packages. The EXAFS spectra were obtained by subtracting the post-edge background from the overall absorption and then normalizing with respect to the edge-jump step. Subsequently, Data of Fe and As K-edge were Fourier transformed to real (*R*) space using a hanning windows (*d*_k_=1.0 Å^-1^) to separate the EXAFS contributions from different coordination shells. To obtain the quantitative structural parameters around central atoms, least-squares curve parameter fitting was performed using the ARTEMIS module of IFEFFIT software packages. The data of As K-edge XANES spectra were fitted using linear combination fitting (LCF) for analyzing the fraction of As(III/V) in SAFeC after adsorption.

# Theoretical calculation method

All density functional theory (DFT) calculations were performed using the Gaussian 16 Rev. C.01 software package. The B3LYP functional combined with Grimme’s D3(BJ) empirical dispersion correction was employed to account for long-range interactions. Geometry optimizations were carried out using the def2-SVP basis set for all atoms. To verify that the optimized geometries correspond to true minima, harmonic vibrational frequency analyses were performed at the same level of theory, confirming the absence of imaginary frequencies. The reaction energy calculations were corrected using the zero-point energy (ZPE) contribution, which improves the accuracy of energy estimations, particularly for systems involving hydrogen atoms. The reaction energy calculations were corrected using the zero-point energy (ZPE) contribution obtained from the frequency analyses, which improves the accuracy of energy estimations, particularly for systems involving hydrogen atoms. The molecular orbital properties of SAFeC including the highest occupied molecular orbital (HOMO), lowest unoccupied molecular orbital (LUMO), density of states (DOS), and partial density of states (PDOS) were analyzed. Weak interactions between SAFeC and As(III) were further explored using the independent gradient model (IGM) method implemented in Multiwfn.

# The calculation of adsorption capacity and removal efficiency.

The adsorption capacity and removal efficiency of SAFeC for As(III) were calculated based on the following equation, respectively.

$$\text{Adsorption capacity (}\text{q}_{\text{e}}\text{, mg·}\text{g}^{\text{-1}}\text{) =}\frac{\text{(}\text{C}_{\text{0}}\text{ -}\text{ C}_{\text{e}}\text{) × }\text{V}}{\text{m}}$$

$$\text{Removal efficiency (\%) =}\frac{\text{(}\text{C}_{\text{0}}\text{ -}\text{ C}_{\text{e}}\text{)}}{\text{C}_{\text{0}}}\text{ × 100\%}$$

where *C*_0_ (mg·L^-1^) is the initial concentration of As(III), and *C_e_* (mg·L^-1^) is the equilibrium concentration of As(III). *m* (g) and *V* (L) represents the mass of SAFeC and the volume of solution, respectively.

# Adsorption kinetic model.

Pseudo-first order model and pseudo-second order model were used to analyze the adsorption kinetic data.

$$\text{Pseudo-first order model: }\text{q}_{\text{t}}\text{ = }\text{q}_{\text{e}}\text{·(1 - }\text{e}^{\text{-}\text{k}_{\text{1}}\text{t}}\text{)}$$

$$\text{Pseudo-second order model: }\text{q}_{\text{t}}\text{ = }\frac{\text{k}_{\text{2}}\text{q}_{\text{e}}^{\text{2}}\text{t}}{\text{1+}\text{k}_{\text{2}}\text{q}_{\text{e}}\text{t}}$$

Where *q_t_* (mg·g^-1^) and *q_e_* (mg·g^-1^) are the adsorbed As(III) amounts at predetermined time (*t*, min) and equilibrium, respectively. *k*_1_ (min^-1^) and *k*_2_ (g·mg^-1^·min^-1^) are the rate constant of the pseudo-first order model and pseudo-second order model, respectively.

# Isotherm adsorption model.

The isotherm adsorption data was analyzed using Langmuir model and Freundlich model.

$$\text{Langmuir model: }\text{q}_{\text{e}}\text{ = }\frac{\text{q}_{\text{m}}\text{K}_{\text{L}}\text{C}_{\text{e}}}{\text{1}\text{+}\text{K}_{\text{L}}\text{C}_{\text{e}}}$$

$$\text{Freundlich model:} \text{q}_{\text{e}}\text{ =}\text{ K}_{\text{F}}\text{C}_{\text{e}}^{\text{n}}$$

where *q_m_* is the maximum adsorption capacity of CSBC for As(III) (mg·g^-1^). *C_e_* (mg·L^-1^) is the As(III) concentration at equilibrium. *K_L_* (L·mg^-1^) is the adsorption affinity constant from Langmuir model. *K_F_* (mg·g^-1^·(mg·L^-1^)^-n^) is the Freundlich constant. *n* is the Freundlich intensity coefficient.

**Supplementary Figures**

**Figure S1.** a) XRD patterns and b) FTIR spectra of crayfish shell.

**Figure S2.** a) Elemental composition and b) Fe content of CXFeY.

**Figure S3.** SEM-EDS map of CXFeY.

**Figure S4.** a) N_2_ adsorption-desorption isotherms and b) pore size distribution of CXFeY.

**Figure S5.** a) BET surface area, b) total pore volume and c) average pore diameter of CXFeY.

**Figure S6.** XRD patterns of CXFeY.

**Figure S7.** Raman spectra of CXFeY.

**Figure S8.** a-c) EPR spectra, d-f) radical spin concentration and g-factor of CXFeY.

**Figure S9.** a-c) FTIR spectra of CXFeY and d-i) corresponding 2D-COS map.

**Figure S10.** C 1s XPS spectra of CXFeY and relative content of various C species.

**Figure S11.** Fe 2p XPS spectra of CXFeY and relative content of various Fe species.

**Figure S12.** O 1s XPS spectra of CXFeY and relative content of various O species.

**Figure S13.** N 1s XPS spectra of CXFeY and relative content of various N species.

**Figure S14.** Zeta potential of CXFeY at different pH values.

**Figure S15.** EIS Nyquist plots of CXFeY.

**Figure S16.** EDC and EAC values of CXFeY and the relative contributions of EDC and EAC to EEC.

**Figure S17.** CV curves and specific capacitance of CXFeY.

**Figure. S18.** Tafel plots, corrosion potential and corrosion current of CXFeY.

**Figure S19.** Fe K-edge EXAFS and fitting results for a) Fe foil, b) FeO, c) Fe_2_O_3_, d) FePc, e) C5Fe0.5, and f) C5Fe2 at *K* space.

**Figure S20.** Fe K-edge EXAFS and fitting results for a) Fe foil, b) FeO, c) Fe_2_O_3_, d) FePc at *R* space.

**Figure S21.** Fe leaching amount during reaction.

**Figure S22.** As(V) concentration and percentage in solution without adding CXFeY during 360 min.

**Figure S23.** EPR spectra of DMPO-·OH/·O_2_^-^ and TEMP-^1^O_2_ in a) CXFeY-As(III) systems without CXFeY and b) CXFeY-As(III) systems without As(III).

**Figure S24.** SEM-EDS maps of CXFeY after reaction with As(III).

**Figure S25.** XRD patterns of CXFeY after reaction with As(III).

**Figure S26.** FTIR spectra of CXFeY before and after reaction with As(III).

**Figure S27.** As 2p XPS spectra of CXFeY before and after reaction, and relative content of various As species after reaction.

**Figure S28.** Fe 2p XPS spectra of CXFeY after reaction and relative content of various Fe species.

**Figure S29.** O 1s XPS spectra of CXFeY after reaction and relative content of various O species.

**Figure S30.** N 1s XPS spectra of CXFeY after reaction and relative content of various N species.

**Figure S31.** As K-edge EXAFS and fitting results for a) As foil, b) As_2_O_3_, c) As_2_O_5_, d) FeAsO_4_, e) C5Fe0.5, and f) C5Fe2 at *K* space.

**Figure S32.** Effect of initial pH on As(III) removal by CYFeX. Reaction conditions: *C*_0_ (As(III)) = 1 mg·L^-1^, CYFeX = 1 g·L^-1^, background electrolyte: 0.01 M NaNO_3_, T = 298.15 K.

**Figure S33.** As(III) removal by CYFeX in different type of water system (river water, tap water, and drinking water). Reaction conditions: *C*_0_ (As(III)) = 1 mg·L^-1^, CYFeX = 1 g·L^-1^, T = 298.15 K.

**Figure S34.** Effect of co-existing anions and ionic strength on As(III) removal by CYFeX. Reaction conditions: C_0_ (As(III)) = 1 mg·L^-1^, CYFeX = 1 g·L^-1^, initial pH = 7, T = 298.15 K.

**Supplementary Tables**

**Table S1.** The assignment and sign of peaks in synchronous and asynchronous (in parentheses) 2D-COS-FTIR maps of C3FeY.

| Position (cm^-1^) | Peak assignment | Sign* | | | | |
| --- | --- | --- | --- | --- | --- | --- |
|  |  | C=O | CO_3_^2-^ | C-O | C-C | Fe-O |
| 1797 | C=O | + | + (-) | - (+) | + (+) | - (+) |
| 1435 | CO_3_^2-^ |  | + | - (+) | + (+) | - (-) |
| 1050 | C-O |  |  | + | - (-) | + (+) |
| 872 | C-C |  |  |  | + | - (+) |
| 570 | Fe-O |  |  |  |  | + |

*: + and - represented positive and negative correlation, respectively.

**Table S2.** The assignment and sign of peaks in synchronous and asynchronous (in parentheses) 2D-COS-FTIR maps of C5FeY.

| Position (cm^-1^) | Peak assignment | Sign* | | | | |
| --- | --- | --- | --- | --- | --- | --- |
|  |  | C=O | CO_3_^2-^ | C-O | C-C | Fe-O |
| 1797 | C=O | + | + (+) | - (-) | + (-) | - (-) |
| 1435 | CO_3_^2-^ |  | + | - (-) | + (-) | - (-) |
| 1050 | C-O |  |  | + | - (+) | + (-) |
| 872 | C-C |  |  |  | + | - (-) |
| 570 | Fe-O |  |  |  |  | + |

*: + and - represented positive and negative correlation, respectively.

**Table S3.** The assignment and sign of peaks in synchronous and asynchronous (in parentheses) 2D-COS-FTIR maps of C7FeY.

| Position (cm^-1^) | Peak assignment | Sign* | | | | |
| --- | --- | --- | --- | --- | --- | --- |
|  |  | C=O | CO_3_^2-^ | C-O | C-C | Fe-O |
| 1797 | C=O | + | + (+) | - (-) | + (+) | - (-) |
| 1435 | CO_3_^2-^ |  | + | + (+) | - (-) | + (+) |
| 1050 | C-O |  |  | + | + (+) | + (+) |
| 872 | C-C |  |  |  | + | + (+) |
| 570 | Fe-O |  |  |  |  | + |

*: + and - represented positive and negative correlation, respectively.

**Table S4.** EXAFS fitting parameters at Fe K-edge for various samples (*Ѕ*_0_^2^ = 0.85).

| Sample | Shell | *CN* ^a^ | *R* (Å) ^b^ | σ^2^ (Å^2^·10^-3^) ^c^ | ΔE_0_ (eV) ^d^ | *R* factor (%) |
| --- | --- | --- | --- | --- | --- | --- |
| Fe foil | Fe-Fe | 8* | 2.471±0.019 | 3.6 | 8.135±3.21 | 0.6 |
| FeO | Fe-O | 6* | 2.122±0.022 | 9.2 | 0.769±0.16 | 0.7 |
|  | Fe-Fe | 12* | 3.077±0.033 | 9.4 | 1.617±0.73 |  |
| Fe_2_O_3_ | Fe-O | 3* | 1.911±0.011 | 4.6 | -4.623±1.08 | 1.4 |
|  | Fe-O | 3* | 2.088±0.012 | 7.9 | -7.623±0.78 |  |
| FePc | Fe-N | 4* | 2.017±0.018 | 10.6 | 8.873±2.03 | 1.5 |
| C5Fe0.5 | Fe-N | 3.6±0.2 | 1.968±0.021 | 7.7 | -5.009±1.14 | 1.2 |
| C5Fe2 | Fe-O/N | 5.1±0.4 | 1.957±0.026 | 8.8 | -8.346±1.29 | 1.1 |

^a^ *CN*, coordination numbers; ^b^ *R*: distance between absorber and backscatter atoms; ^c^ *σ*^2^: Debye-Waller factors to account for both thermal and structural disorders; ^d^ Δ*E*_0_, inner potential correction. *R* factor indicates the goodness of the fit.

**Table S5.** Kinetic parameters for As adsorption by CXFeY.

| Treatment | Pseudo-first order | | | Pseudo-second order | | |
| --- | --- | --- | --- | --- | --- | --- |
|  | *q_e_*  (mg·g^-1^) | *k*_1_  (min^-1^) | R^2^ | *q_e_*  (mg·g^-1^) | *k*_2_  (g·(mg·min)^-1^) | R^2^ |
| C3-As(III) | 1.93 | 0.0228 | 0.9846 | 2.28 | 0.0115 | 0.9941 |
| C3Fe0.5-As(III) | 36.88 | 0.0689 | 0.9935 | 40.39 | 0.0025 | 0.9855 |
| C3Fe1-As(III) | 36.62 | 0.0737 | 0.9887 | 39.90 | 0.0027 | 0.9910 |
| C3Fe2-As(III) | 35.71 | 0.0607 | 0.9725 | 39.24 | 0.0022 | 0.9808 |
| C3Fe5-As(III) | 35.73 | 0.0658 | 0.9917 | 39.57 | 0.0022 | 0.9811 |
| C5-As(III) | 1.91 | 0.0212 | 0.9850 | 2.27 | 0.0105 | 0.9971 |
| C5Fe0.5-As(III) | 30.80 | 0.0793 | 0.9903 | 33.56 | 0.0035 | 0.9833 |
| C5Fe1-As(III) | 29.81 | 0.0908 | 0.9862 | 32.43 | 0.0041 | 0.9941 |
| C5Fe2-As(III) | 30.08 | 0.1024 | 0.9857 | 32.64 | 0.0045 | 0.9843 |
| C5Fe5-As(III) | 28.76 | 0.0682 | 0.9803 | 31.77 | 0.0030 | 0.9900 |
| C7-As(III) | 1.52 | 0.0286 | 0.9843 | 1.75 | 0.0203 | 0.9873 |
| C7Fe0.5-As(III) | 24.66 | 0.0163 | 0.9893 | 30.40 | 0.0005 | 0.9770 |
| C7Fe1-As(III) | 6.05 | 0.0365 | 0.9155 | 6.79 | 0.0075 | 0.9652 |
| C7Fe2-As(III) | 5.80 | 0.0336 | 0.9953 | 6.64 | 0.0063 | 0.9901 |
| C7Fe5-As(III) | 4.50 | 0.0155 | 0.9965 | 5.56 | 0.0028 | 0.9968 |

**Table S6.** Isotherm constants for As adsorption by CXFeY.

| Treatment | Langmuir | | | Freundlich | | |
| --- | --- | --- | --- | --- | --- | --- |
|  | *K_L_*  (L·mg^-1^) | *q_m_*  (mg·g^-1^) | R^2^ | *K_F_*  (mg·g^-1^·(mg·L^-1^)^-n^) | *n* | R^2^ |
| C3-As(III) | 0.0212 | 4.85 | 0.9952 | 0.2972 | 0.5216 | 0.9897 |
| C3Fe0.5-As(III) | 0.0312 | 56.70 | 0.9849 | 4.2856 | 0.5174 | 0.9539 |
| C3Fe1-As(III) | 0.0280 | 88.87 | 0.9855 | 5.7029 | 0.5563 | 0.9704 |
| C3Fe2-As(III) | 0.0248 | 131.53 | 0.9970 | 5.8381 | 0.6492 | 0.9878 |
| C3Fe5-As(III) | 0.0306 | 185.36 | 0.9928 | 8.4485 | 0.6917 | 0.9857 |
| C5-As(III) | 0.0494 | 3.25 | 0.9708 | 0.4704 | 0.3828 | 0.9949 |
| C5Fe0.5-As(III) | 0.0272 | 212.44 | 0.9889 | 7.9229 | 0.7358 | 0.9735 |
| C5Fe1-As(III) | 0.0313 | 214.10 | 0.9814 | 8.9951 | 0.7290 | 0.9672 |
| C5Fe2-As(III) | 0.0369 | 223.40 | 0.9929 | 11.6089 | 0.7029 | 0.9957 |
| C5Fe5-As(III) | 0.0193 | 237.50 | 0.9937 | 6.5093 | 0.7619 | 0.9883 |
| C7-As(III) | 0.0468 | 2.40 | 0.9691 | 0.3259 | 0.3945 | 0.9943 |
| C7Fe0.5-As(III) | 0.0326 | 57.11 | 0.9820 | 4.7563 | 0.4984 | 0.9619 |
| C7Fe1-As(III) | 0.0253 | 19.40 | 0.9715 | 1.2897 | 0.5199 | 0.9251 |
| C7Fe2-As(III) | 0.0279 | 13.61 | 0.9835 | 1.0661 | 0.4899 | 0.9777 |
| C7Fe5-As(III) | 0.0875 | 9.40 | 0.9603 | 1.9951 | 0.3211 | 0.9594 |

**Table S7.** Comparison of As(III) adsorption capacity with currently reported Fe-loading adsorbent.

| Adsorbents | Fe loading  (wt %) | *q_max_*  (mg·g^-1^) | Normalized *q_max_*  (mg·(g Fe)^-1^) | Ref. |
| --- | --- | --- | --- | --- |
| M-GO | 54.60 | 85.00 | 155.68 | [1] |
| Fe-NN/BFs | 8.68 | 6.07 | 69.93 | [2] |
| FeBC900 | 7.50 | 48.57 | 647.60 | [3] |
| nZVI-B500 | 20.00 | 74.30 | 371.50 | [4] |
| Fe_3_C@BC-C | 26.50 | 21.90 | 82.64 | [5] |
| BC-ZVI | 28.10 | 129.24 | 459.93 | [6] |
| Fe@MOF-808 | 8.55 | 142.82 | 1670.41 | [7] |
| Co_0.5_Fe_2.5_O_4_ | 35.80 | 84.56 | 236.20 | [8] |
| ChitFe7 | 14.50 | 35.25 | 243.10 | [9] |
| Hum/HTC | 12.60 | 189.40 | 1503.17 | [10] |
| MLF-NS | 30.40 | 36.95 | 121.55 | [11] |
| **C3F0.5** | **0.51** | **56.70** | **11107.28** | **This work** |
| **C3F1** | **1.04** | **88.87** | **8575.74** |  |
| **C3F2** | **2.06** | **131.53** | **6398.87** |  |
| **C3F5** | **5.19** | **185.36** | **3573.55** |  |
| **C5F0.5** | **0.53** | **212.44** | **39833.54** |  |
| **C5F1** | **1.03** | **214.10** | **20760.15** |  |
| **C5F2** | **2.07** | **223.40** | **10771.91** |  |
| **C5F5** | **5.25** | **237.50** | **4520.47** |  |
| **C7F0.5** | **0.39** | **57.11** | **14469.49** |  |
| **C7F1** | **0.96** | **19.40** | **2023.33** |  |
| **C7F2** | **2.06** | **13.61** | **659.80** |  |
| **C7F5** | **4.06** | **9.40** | **231.51** |  |

**Table S8.** EXAFS fitting parameters at As K-edge for various samples (*Ѕ*_0_^2^=0.87).

| Sample | Shell | *CN* ^a^ | *R* (Å) ^b^ | σ^2^ (Å^2^·10^-3^) ^c^ | ΔE_0_ (eV) ^d^ | *R* factor (%) |
| --- | --- | --- | --- | --- | --- | --- |
| As Foil | As-As | 3* | 2.52±0.012 | 0.0017 | 5.87±1.18 | 0.93 |
| As_2_O_3_ | As-O | 3.0±0.2 | 1.62±0.028 | 0.0021 | 1.26±0.57 | 1.06 |
|  | As-As | 0.9±0.1 | 2.91±0.032 | 0.0182 |  |  |
| FeAsO_4_ | As-O | 1.9±0.3 | 1.72±0.014 | 0.0045 | 6.62±1.62 | 1.21 |
|  | As-Fe | 1.4±0.5 | 2.96±0.027 | 0.0253 |  |  |
| As_2_O_5_ | As-O | 3.7±0.4 | 1.69±0.011 | 0.0013 | 7.39±2.01 | 1.16 |
| C5Fe0.5 | As-O | 2.2±0.2 | 1.81±0.012 | 0.0011 | 8.22±1.63 | 0.98 |
| C5Fe2 | As-O | 2.1±0.5 | 1.76±0.026 | 0.0011 | 3.59±1.35 | 1.38 |
|  | As-Fe | 1.2±0.2 | 2.92±0.023 | 0.0027 |  |  |

^a^ *CN*, coordination number; ^b^ *R*, distance between absorber and backscatter atoms; ^c^ *σ*^2^, Debye-Waller factor to account for both thermal and structural disorders; ^d^ *ΔE*_0_, inner potential correction; *R* factor indicates the goodness of the fit.

# References

1.	Y. Yoon, W. K. Park, T.-M. Hwang, D. H. Yoon, W. S. Yang, and J. W. Kang, “Comparative evaluation of magnetite–graphene oxide and magnetite-reduced graphene oxide composite for As(III) and As(V) removal,” *Journal of Hazardous materials* 304 (2016): 196-204, https://doi.org/10.1016/j.jhazmat.2015.10.053.

2.	Y. Wei, S. Wei, C. Liu, T. Chen, Y. Tang, J. Ma, et al., “Efficient removal of arsenic from groundwater using iron oxide nanoneedle array-decorated biochar fibers with high Fe utilization and fast adsorption kinetics,” *Water Research* 167 (2019): 115107, https://doi.org/10.1016/j.watres.2019.115107.

3.	Y. Xu, X. Xie, Y. Feng, M. A. Ashraf, Y. Liu, C. Su, et al., “As(III) and As(V) removal mechanisms by Fe-modified biochar characterized using synchrotron-based X-ray absorption spectroscopy and confocal micro-X-ray fluorescence imaging,” *Bioresource Technology* 304 (2020): 122978, https://doi.org/10.1016/j.biortech.2020.122978.

4.	K. Liu, F. Li, X. Zhao, G. Wang, and L. Fang, “The overlooked role of carbonaceous supports in enhancing arsenite oxidation and removal by nZVI: Surface area *versus* electrochemical property,” *Chemical Engineering Journal* 406 (2021): 126851, https://doi.org/10.1016/j.cej.2020.126851.

5.	N. Zhao, X. Tan, J. Xiong, N. Chen, J. Gao, R. Wang, et al., “Quantitative analysis on the redox conversion mechanism of Cr(VI) and As(III) by iron carbide based biochar composites,” *Chemical Engineering Journal* 446 (2022): 137417, https://doi.org/10.1016/j.cej.2022.137417.

6.	L. Xu, Z. Shu, L. Feng, J. Zhou, T. Li, Z. Zhao, et al., “Fresh biomass derived biochar with high-load zero-valent iron prepared in one step for efficient arsenic removal,” *Journal of Cleaner Production* 352 (2022): 131616, https://doi.org/10.1016/j.jclepro.2022.131616.

7.	W. Li, Z. Liu, L. Wang, G. Gao, H. Xu, W. Huang, et al., “FeS_x_@MOF-808 composite for efficient As(III) removal from wastewater: behavior and mechanism,” *Journal of Hazardous materials* 446 (2022): 130681, https://doi.org/10.1016/j.jhazmat.2022.130681.

8.	Z. Lin, F. Deng, W. Ren, Z. Wang, X. Xiao, P. Shao, et al., “Integration of adsorption and simultaneous heterogeneous catalytic oxidation by defective CoFe_2_O_4_ activated peroxymonosulfate for efficient As(III) removal: Performance and new insight into the mechanism,” *Chemical Engineering Journal* 454 (2023): 139960, https://doi.org/10.1016/j.cej.2022.139960.

9.	J. Joseph, A. Väisänen, A. B. Patil, and M. Lahtinen, “The effect of synthesis conditions on the in situ grown MIL-100(Fe)-chitosan beads: Interplay between structural properties and arsenic adsorption,” *Journal of Hazardous materials* 463 (2024): 132893, https://doi.org/10.1016/j.jhazmat.2023.132893.

10.	L. Cheng, Z. Lu, J. Liu, J. Liu, Y. Zhao, Z. Ni, et al., “Novel heterogeneous Fenton catalysts for promoting carbon iron electron transfer by one-step hydrothermal synthesization,” *Journal of Colloid and Interface Science* 656 (2024): 597-608, https://doi.org/10.1016/j.jcis.2023.11.150.

11.	X. Wang, J. Zhu, L. Qi, Y. Qin, B. Ren, and X. Wang, “Co-sorption of arsenic and fluoride using the hierarchical Mg-La-Fe nanosheets derived from layered double oxides,” *Chemical Engineering Journal* 499 (2024): 156340, https://doi.org/10.1016/j.cej.2024.156340.
